# Integrative blood-based characterization of oxidative mitochondrial DNA damage variants implicates Mexican American’s metabolic risk for developing Alzheimer’s disease

**DOI:** 10.1038/s41598-023-41190-6

**Published:** 2023-09-07

**Authors:** Danielle Marie Reid, Robert C. Barber, Harlan P. Jones, Roland J. Thorpe, Jie Sun, Zhengyang Zhou, Nicole R. Phillips

**Affiliations:** 1 Microbiology, Immunology, and Genetics, School of Biomedical Sciences, UNT Health Science Center, Fort Worth, TX USA; 2 Family Medicine, Texas College of Osteopathic Medicine, UNT Health Science Center, Fort Worth, TX USA; 3 Institue for Translational Research, UNT Health Science Center, Fort Worth, TX USA; 4 Johns Hopkins Center for Health Disparities Solutions, Johns Hopkins Bloomberg School of Public Health, Baltimore, MD USA; 5 Biostatistics and Epidemiology, School of Public Health, UNT Health Science Center, Fort Worth, TX USA

**Keywords:** Molecular biology, Mutation, Sequencing, Metabolic disorders, Neurological disorders

## Abstract

Alzheimer’s Disease (AD) continues to be a leading cause of death in the US. As the US aging population (ages 65 +) expands, the impact will disproportionately affect vulnerable populations, e.g., Hispanic/Latino population, due to their AD-related health disparities. Age-related regression in mitochondrial activity and ethnic-specific differences in metabolic burden could potentially explain in part the racial/ethnic distinctions in etiology that exist for AD. Oxidation of guanine (G) to 8-oxo-guanine (8oxoG) is a prevalent lesion and an indicator of oxidative stress and mitochondrial dysfunction. Damaged mtDNA (8oxoG) can serve as an important marker of age-related systemic metabolic dysfunction and upon release into peripheral circulation may exacerbate pathophysiology contributing to AD development and/or progression. Analyzing blood samples from Mexican American (MA) and non-Hispanic White (NHW) participants enrolled in the Texas Alzheimer’s Research & Care Consortium, we used blood-based measurements of 8oxoG from both buffy coat PBMCs and plasma to determine associations with population, sex, type-2 diabetes, and AD risk. Our results show that 8oxoG levels in both buffy coat and plasma were significantly associated with population, sex, years of education, and reveal a potential association with AD. Furthermore, MAs are significantly burdened by mtDNA oxidative damage in both blood fractions, which may contribute to their metabolic vulnerability to developing AD.

## Introduction

Alzheimer’s disease (AD) is the most common form of dementia, characterized by symptoms of cognitive decline such as memory deficits, impaired problem-solving, difficulty communicating, and additional cerebral incompetencies^1,2^. This heterogenous neurodegenerative disease is commonly known for its neurotoxic pathophysiological properties including the accumulation of amyloid beta (Aβ) plaques and tangles of hyperphosphorylated tau protein^1–3^. However, impaired mitochondrial function and chronic inflammation are frequently reported and can be considered as contributors to the observed endophenotypic manifestations of cognitive impairment (CI) likely caused by Alzheimer’s^2–8^. Particularly, non-Hispanic Whites (NHWs) appear to exhibit an inflammatory endophenotype^4–7^, while Mexican Americans (MAs) present a metabolic endophenotype^5^. This evidence may point to population-specific biological and environmental factors influencing CI. Importantly, current established biomarkers are invasive, expensive, and have limited accessibility (i.e., cerebral spinal fluid testing, neuroimaging). Identifying blood-based biomarkers capable of predicting disease onset and assessing disease progression (e.g., preclinical AD, mild cognitive impairment [MCI], and AD) is of great importance to understand the pathophysiological heterogeneity of AD which will inform ultimately more precise therapeutics.

Type-2 diabetes (T2D) is a considerable risk factor for AD due to its comorbid association with CI; however, the precise pathophysiological mechanisms connecting these two complex diseases are unclear. As the aging Hispanic/Latino population is expected to exponentially increase compared to other ethnic/racial groups, the healthcare burden affecting this population is expected to worsen due to age-related diseases (e.g., AD and diabetes)^1,9^. Between the year 2018 to 2040, the number of older Hispanic adults is expected to increase by 175% compared to older Black adults with an anticipated 88% increase in population size^10^, and from 2012 to 2060, the Latino aging population is estimated to increase from 3 million to 21.5 million individuals in the U.S. with an anticipated increase of Latino individuals with AD by 832%^9^. It has been previously reported that the Ser(326)Cys polymorphism (rs1052133) in 8-Oxoguanine glycosylase (OGG1*)*, an important glycosylase of the base excision repair pathway involved in the recognition and excision of 8oxoG within DNA, delays repair of oxidative DNA damage^11^ and is associated with T2D risk in MAs^13^. Recent observations suggest OGG1 regulates cellular energy metabolism and thus shapes metabolic phenotypes during high-fat diet exposure, indicating DNA damage repair may be important to metabolic health^12–14^. Studies by Komakula et al., and Sampath et al., revealed that functional OGG1 prevented obesity and metabolic dysfunction upon induction in response to a high-fat diet potentially via direct or indirect expression changes of *PGC-1α* and fatty acid oxidation ^12,13^. Similarly, reduced expression of *PGC-1α* is consistently observed in T2D patients^15,16^ and is linked to elevated ROS levels and decreased levels of β-oxidation enzymes^17^. AD disproportionately affects Hispanics/Latinos compared to NHWs (e.g., one study indicates 14% of aging Hispanics have AD compared to 10% of aging NHWs^18^, and others report that aging Hispanics are about 1.5 times as likely to have AD compared to aging NHWs^19–21^); this is thought to be due to the increased prevalence of metabolic syndrome, obesity, cardiovascular health risks, and diabetes^9,22,23^. These observations combined with our previous data indicating MAs have elevated levels of oxidative damage compared to NHWs, leads us to the premise that mitochondrial health may hold greater biological importance in the development of age-related disease for individuals with Hispanic/Latino ancestry^24^.

Compared to the nuclear genome, mitochondrial DNA is especially susceptible to oxidative damage. Elevated levels of ROS generate oxidative stress (OS) and an oxidative environment capable of oxidatively damaging important biomolecules and may cause detrimental effects in genomic coding regions^25–27^. Due to the low oxidation potential of guanine, the most common forms of oxidative DNA damage are: 8-oxo-7,8-dihydroguanine (8oxoG) and 8-oxo-7,8-dihydrodeoxyguanine (8oxodG)^26–31^. Oxidation of guanine possesses unique mutagenic properties that when left unrepaired, can perturb cellular function through several mechanisms and effect protein-DNA binding (e.g., transcription factors)^26,27,30,31^. Furthermore, oxidized guanine can result in missense mutations and often modify the activity of downstream products, such as RNAs and proteins^25–27,30,32^. Due to the nature of the mitochondrial genome within various cell types and tissues, oxidative DNA damage will influence distinctive pathophysiological outcomes in tissue with differing function depending on the location, metabolic activity, and enzymatic processes^31^. In response to mitochondrial damage there are QC processes at the cellular, organellar, and molecular level that are employed to preserve mitochondrial integrity^33^. As mitochondria work in dynamic networks, fusion with nearby mitochondria can recover function; however, excessive damage undermines fusion activity resulting in fragmented mitochondria that are removed through mitophagy and/or apoptosis^33^.

Growing evidence implicates oxidative DNA damage as a primary and secondary contributor to pathology observed in the AD continuum^34–37^. Recent evidence from our lab analyzing blood-based indices of mtDNA copy number (CN) and cell-free mtDNA (cf-mtDNA) to investigate mitochondrial dysfunction in complex disease (T2D and CI) among MAs showed that mtDNA CN was significantly associated with both T2D and CI^23^. Also, cf-mtDNA was found to be higher in individuals with either disease, reaching significant levels in individuals with both diseases compared to normal controls^23^. Cellular mtDNA CN is an indicator of mitochondrial biogenesis and cellular energetics^23,38^, which can be used as a measurement of mitochondrial health^39^.

Circulating cell-free mitochondrial DNA (ccf-mtDNA) has been increasingly studied as a biomarker for systemic inflammation during cellular stress or apoptosis. In this process, mtDNA fragments are released by cells into the bloodstream, where their bacterial origins cause them to be recognized as a damage associated molecular pattern (DAMP), thereby eliciting an inflammatory response through activation of innate immune cells^23,39–41^. There are numerous studies assessing ccf-mtDNA as a clinical diagnostic and predictive biomarker (e.g., in affective disorders, and mitochondrial, autoimmune, neurological, and cardiovascular diseases)^39,40,42–45^, and accumulating evidence indicates that mitochondrial dysfunction and mtDNA damage are correlated with disease severity and levels of ccf-mtDNA^40,44^.

Our mtDNA CN and cf-mtDNA data can be considered as a proxy for our more recent data evaluating mitochondrial 8oxoG variant load since it does not capture if mutation has occurred. More recently in a similar population-based cohort, we discovered that mtDNA variants indicative of 8oxoG were significantly elevated in MAs compared to NHWs and was associated with sex, education, and suggestive for cognitive function^24^. Correspondingly, Miller, et al., revealed AD neurons compared to age-matched controls had significantly elevated levels of somatic single nucleotide variants (sSNVs) than anticipated when considering sSNVs are known to increase with age^37^, and the distribution of the variants are presumed to occur secondary to developing disease pathology^37^. Further analysis established potential mechanisms of oxidative DNA damage developed from 8oxoG (nonsynonymous mutations, e.g., C > A) that might contribute to the significant increase of sSNVs in AD, especially in protein-coding genes^37^. The increased substitution mutations could be a result of increased ROS and OS, a common feature observed in AD brains, the CNS, and periphery, which can contribute to inflammation and mitochondrial dysfunction^37,46^. This accumulating evidence may point at a mutational signature important to AD pathophysiology influencing the differing endophenotype reported in AD, particularly of those with different mitochondrial capacity, metabolic health, and comorbidity risk such as those linked to ethnic/racial AD health disparities.

Oxidative transversion substitution mutations may be associated with increased mitochondrial dysfunction due to impaired mitochondrial capacity, metabolic health, and/or lifestyle affecting mitochondrial health, and contribute to the continuous progression of AD until a clinical endpoint, death. In this current study, our objective was to use a blood-based measurement of 8oxoG sSNVs as an indicator of impaired mitochondrial function to investigate the role of mitochondria in pathophysiology of complex disease by (1) characterizing associations to population and sex, (2) highlighting burdened genomic regions that influence mitochondrial function, and (3) determining differences in buffy coat or plasma on evaluating AD risk and/or endophenotype.

## Results

Descriptive statistics for the cohort analyzed for cellular mitochondrial 8oxoG variants are displayed in Table 1. As anticipated, MMSE, CDR sum, and years of education in both populations had significantly different means based on cognitive status. Age significantly differed in MAs between cognitive groups and years of education was lower compared to NHWs.

**Table 1:** Descriptive statistics of NHW and MA participants categorized by population and cognitive phenotype in the Texas Alzheimer’s Research and Care Consortium for buffy coat mitochondrial DNA oxidative mutational load.

	NC		MCI	AD	*P*-value^a^
Total Number of Subjects	328		127	104	
Non-Hispanic Whites	153		43	64	
Age [CI]	70.39 ± 1.178		71.35 ± 1.421	71.70 ± 1.056	0.338
Sex (F) [n, %]	78, 50.98%		21, 48.84%	29, 45.31%	0.747
Mini Mental State Exam (MMSE) [CI]	29.11 ± 0.1759		27.63 ± 0.6223	21.53 ± 1.413	< 0.001^b^
Clinical Dementia Rating (CDR) Sum [CI]	0.007 ± 0.009		1.163 ± 0.2181	5.344 ± 0.8515	< 0.001^c^
Years of Education [CI]	16.07 ± 0.4063		14.56 ± 0.6597	15.11 ± 0.7524	0.001^d^
BMI kg/m^2^ [CI]	27.331 ± 1.150		27.272 ± 2.328	27.394 ± 1.062	0.996
Diabetes (Y) [n, %]	59, 38.56%		18, 41.86%	22, 34.38%	0.723
Hypercholesterolemia (Y) [n, %]	90, 58.82%		26, 60.47%	50, 78.13%	0.023
Hyperlipidemia (Y) [n, %]	56, 36.60%		15, 34.88%	39, 60.94%	0.002
Hypertension (Y) [n, %]	100, 65.36%		31, 72.09%	44, 68.75%	0.680
Obesity (Y) [n, %]	27, 17.65%		8, 18.69%	10, 15.63%	0.910
Depression (Y) [n, %]	12, 7.84%		6, 13.95%	17, 26.56%	0.001
Tobacco Abuse (Y) [n, %]	51, 33.33%		18, 41.86%	28, 43.75%	0.279
Alcohol Abuse (Y) [n, %]	3, 1.96%		5, 11.63%	3, 4.69%	0.020
Mexican Americans	175		84	40	
Age [CI]	67.62 ± 0.8156		69.88 ± 1.691	73.37 ± 2.485	< 0.001^e^
Sex (F) [n, %]	99, 56.57%		40, 47.62%	24, 60.00%	0.302
Mini Mental State Exam (MMSE) [CI]	28.14 ± 0.2889		24.93 ± 1.140	19.87 ± 1.860	< 0.001^f^
Clinical Dementia Rating (CDR) Sum [CI]	0.006 ± 7.897_10_^–3^		1.113 ± 0.2261	5.737 ± 1.183	< 0.001^g^
Years of Education [CI]	11.05 ± 0.6598		8.77 ± 1.664	9.75 ± 1.547	0.002^h^
BMI kg/m^2^ [CI]	30.917 ± 0.9992		31.295 ± 2.228	28.717 ± 1.651	0.116
Diabetes (Y) [n, %]	79, 45.14%		32, 38.10%	19, 47.50%	0.484
Hypercholesterolemia (Y) [n, %]	103, 58.86%		52, 61.90%	23, 57.50%	0.862
Hyperlipidemia (Y) [n, %]	89, 50.86%		35, 41.67%	12, 30.00%	0.120
Hypertension (Y) [n, %]	120, 68.57%		63, 75.00%	28, 70.00%	0.567
Obesity (Y) [n, %]	84, 48.00%		38, 45.24%	8, 20.00%	0.005
Depression (Y) [n, %]	19, 10.86%		29, 34.52%	15, 37.50%	< 0.001
Tobacco Abuse (Y) [n, %]	82, 46.86%		39, 46.43%	18, 45.00%	0.978
Alcohol Abuse (Y) [n, %]	6, 3.43%		0, 0.00%	2, 5.00%	0.172

*NC* normal control, *MCI* mild cognitive impairment, *AD* Alzheimer’s Disease.

^a^The mean difference is significant at 0.05.

^b^NC vs. MCI 0.016, NC vs. AD < 0.001, MCI vs. AD < 0.001.

^c^NC vs. MCI <0.001, NC vs. AD < 0.001, MCI vs. AD < 0.001.

^d^NC vs. MCI 0.003, NC vs. AD 0.042, MCI vs. AD 0.542.

^e^NC vs. MCI 0.028, NC vs. AD < 0.001, MCI vs. AD 0.017.

^f^NC vs. MCI <0.001, NC vs. AD < 0.001, MCI vs. AD < 0.001.

^g^NC vs. MCI <0.001, NC vs. AD < 0.001, MCI vs. AD < 0.001.

^h^NC vs. MCI 0.001, NC vs. AD 0.273, MCI vs. AD 0.540.

Directly genotyped and imputed *APOE* frequencies for each population is shown in Supplementary Table 1. Genotype frequencies for *APOE* and *OGG1* by cognitive status in each population are shown in Supplementary Table 2. Hardy–Weinberg proportions for *APOE* and *OGG1* in both populations separately and together indicates the genotype frequencies are in Hardy–Weinberg equilibrium and both the observed and expected genotype frequencies are not significantly different (Supplementary Tables 3 and 4).

### Evaluation of 8oxoG variant count in the buffy coat of MA and NHW TARCC participants

In the MA population 8oxoG variant count was significantly reduced for subjects reporting depression compared to those without depression; mean = 6.548 and 7.704, respectively (Supplemental Fig. 1). Tobacco abuse demonstrated an approach for significance in association with 8oxoG demonstrating a higher variant load compared to non-smokers in MAs; mean = 7.935 and 7.048, respectively (Supplemental Fig. 2). These trends were not observed in the NHW cohort.

Multiple linear regression model predictions in the whole cohort were performed to assess the associations with 8oxoG and to determine if there are predictive interactions. Sex regarding females (*P* = 0.0007), years of education (*P* = 0.0055), BMI (*P* = 0.0288), and tobacco abuse (*P* = 0.0086) were significantly associated with 8oxoG variant count, and the population-sex interaction demonstrated a significant interaction effect (*P* = 0.0038) (Supplementary Table 5). Further analysis of 8oxoG variant count in both population and sex via two-way ANOVA indicates population is significantly associated (*P* < 0.0001), while sex was marginally significant (*P* = 0.0922).

In the subsequent multiple linear regression model, a diabetes × cognition interaction was evaluated and showed a significant association with population (*P* < 0.0001), sex (*P* = 0.0429), years of education (*P* = 0.0109), BMI (*P* = 0.0254), and tobacco abuse (*P* = 0.0155); although the interaction effect was not significant (Supplementary Table 6). Cognitive status with respect to AD displayed a suggestive association compared to controls (*P* = 0.0556).

Previously derived 8oxoG variant load for each subject corresponding to 8oxoG “hotspots”^24^ were further analyzed via multiple linear regression prediction models assessing a population × sex and years of education interaction effect, and a diabetes × cognition interaction effect was less informative than analyzing total 8oxoG variant count (Supplementary Tables 7 and 8). Population stratification lost significant statistical associations (Supplementary Tables 9 and 10) that were observed in Supplementary Table 7 and 8.

Multiple linear regression modelling in MAs indicated that total 8oxoG was significantly associated with cognitive status, sex, years of education, and tobacco abuse (Table 2). BMI did not show significant association in MAs as compared to the regression models investigating interactive effects (Supplementary Tables 5–6). Modelling within NHWs did not demonstrate any associations (Table 3).

**Table 2:** Multiple linear regression prediction model in the Mexican American population considering total cellular 8oxoG variant count. Italics and bolding indicate a *P*-value of significance, while italics alone indicate a p-value approaching significance.

Variable	Coefficient	SE	t-statistic	*P*-value
Constant	5.38624	3.66547	1.469	0.143088
Cognitive Status (with respect to AD)	1.88775	0.90381	2.089	***0.037847***
Cognitive Status (with respect to MCI)	1.11683	0.71666	1.558	0.120533
Sex (with respect to Male)	− 2.23145	0.62169	− 3.589	***0.000406***
Age	− 0.01812	0.04432	− 0.409	0.68306
Years of Education	0.15433	0.06272	2.461	***0.01461***
BMI	0.0733	0.05051	1.451	0.148079
Diabetes (with respect to "Yes")	− 0.97291	0.62086	− 1.567	0.118495
Depression (with respect to "Yes")	− 1.4407	0.79867	− 1.804	*0.072569*
Tobacco Abuse (with respect to "Yes")	1.97348	0.60289	3.273	***0.001228***
APOE	0.18362	0.65285	0.281	0.778769
OGG1	− 0.03107	0.42738	− 0.073	0.942111
R-squared	0.1199		*P*-value	0.001776
Adjusted R-squared	0.07743		df	11 and 228
F-statistic	2.824		Sample n	201

**Table 3:** Multiple linear regression prediction model in non-Hispanic Whites considering total cellular 8oxoG variant count.

Variable	Coefficient	SE	t-statistic	*P*-value
Constant	0.42632	3.39267	0.126	0.9
Cognitive Status (with respect to AD)	− 0.1306	0.57357	− 0.228	0.82
Cognitive Status (with respect to MCI)	0.23	0.64941	0.354	0.724
Sex (with respect to Male)	0.60026	0.45481	1.32	0.189
Age	0.04559	0.03686	1.237	0.218
Years of Education	0.0119	0.08375	0.142	0.887
BMI	0.07021	0.04965	1.414	0.159
Diabetes (with respect to "Yes")	− 0.17897	0.53328	− 0.336	0.738
Depression (with respect to "Yes")	0.01429	0.64799	0.022	0.982
Tobacco Abuse (with respect to "Yes")	− 0.14434	0.46294	− 0.312	0.756
APOE	0.17776	0.36646	0.485	0.628
OGG1	− 0.28863	0.37952	− 0.761	0.448
R-squared	0.04111		*P*-value	0.7794
Adjusted R-squared	− 0.02168		df	11 and 168
F-statistic	0.6547		Sample n	180

Additional prediction modelling used cognitive status as a binary variable to combine the effects of AD and MCI compared to NCs and showed similar results to the models with greater resolution on cognitive status (Supplementary Tables 11–18).

### Assessment of ccf-mtDNA 8oxoG variant count in MA and NHW TARCC participants

The subset of participants included for the ccf-mtDNA 8oxoG variants were selected from subjects included in the buffy coat analysis to compare the blood fractions collected from the same visit (Table 4). Age, sex, and years of education were considered confounding variables for CI and were utilized with the aim to pairwise match AD with NCs to help reduce the risk of confounders influencing false associations to AD due to the smaller sample size.

**Table 4:** Descriptive statistics of NHW and MA TARCC participants classified by population and cognitive phenotype for plasma (i.e., ccf) mitochondrial DNA oxidative mutational load.

	NC	AD	*P*-value^a^
Total Number of Subjects	63	59	
Non-Hispanic Whites	33	32	
Age [CI]	72.30 ± 3.112	72.19 ± 1.913	0.951
Sex (F) [n, %]	19, 57.58%	19, 58.62%	0.883
Mini Mental State Exam (MMSE) [CI]	28.91 ± 0.4296	20.41 ± 2.214	< 0.001
Clinical Dementia Rating (CDR) Sum [CI]	0.000 ± 0.000	5.625 ± 1.353	< 0.001
Years of Education [CI]	14.12 ± 0.9178	14.13 ± 0.9549	0.996
BMI kg/m^2^ [CI]	27.997 ± 2.291	27.709 ± 1.632	0.842
Diabetes (Y) [n, %]	15, 45.45%	16, 50.00%	0.714
Hypercholesterolemia (Y) [n, %]	17, 51.52%	27, 84.38%	0.005
Hyperlipidemia (Y) [n, %]	11, 33.33%	24, 75.00%	< 0.001
Hypertension (Y) [n, %]	23, 69.70%	22, 68.75%	0.934
Obesity (Y) [n, %]	25, 75.76%	26, 81.25%	0.590
Depression (Y) [n, %]	6, 18.18%	12, 37.50%	0.082
Tobacco Abuse (Y) [n, %]	13, 39.39%	16, 50.00%	0.390
Alcohol Abuse (Y) [n, %]	1, 3.03%	2, 6.25%	0.536
Mexican Americans	30	27	
Age [CI]	73.23 ± 1.971	73.89 ± 3.184	0.733
Sex (F) [n, %]	17, 56.67%	14, 51.85%	0.716
Mini Mental State Exam (MMSE) [CI]	27.83 ± 0.6846	19.63 ± 2.111	< 0.001
Clinical Dementia Rating (CDR) Sum [CI]	0.017 ± 0.03267	5.574 ± 1.282	< 0.001
Years of Education [CI]	9.93 ± 1.971	9.63 ± 1.794	0.824
BMI kg/m^2^ [CI]	31.06 ± 2.166	28.319 ± 1.8647	0.065
Diabetes (Y) [n, %]	12, 40.00%	13, 48.15%	0.536
Hypercholesterolemia (Y) [n, %]	17, 56.67%	17, 62.96%	0.629
Hyperlipidemia (Y) [n, %]	13, 43.33%	8, 29.63%	0.284
Hypertension (Y) [n, %]	21, 70.00%	18, 66.67%	0.787
Obesity (Y) [n, %]	17, 56.67%	23, 85.19%	0.019
Depression (Y) [n, %]	3, 10.00%	11, 40.74%	0.007
Tobacco Abuse (Y) [n, %]	12, 40.00%	13, 48.15%	0.536
Alcohol Abuse (Y) [n, %]	1, 3.33%	2, 7.41%	0.492

^a^The mean difference is significant at 0.05.

Genotype frequencies obtained and imputed for *APOE* in each population and genotype frequencies for *APOE* and *OGG1* distributed by cognitive status in each population are shown (Supplementary Table 19 and 20). Testing for Hardy–Weinberg Equilibrium for *APOE* and *OGG1* were insignificant for the total cohort and within each population, indicating that the genotype frequencies are in equilibrium (Supplementary Tables 21 and 22).

Although our attempt to match samples based on age was unsuccessful; a Pearson’s correlation was performed to exclude age as a potential cofounder and showed age does not need to be considered a covariate in our dataset as it was not correlated with total ccf-mtDNA 8oxoG variant count (Supplementary Fig. 3).

### MAs, especially females, have a greater burden in total ccf-8oxoG variant count

**Figure 1:**
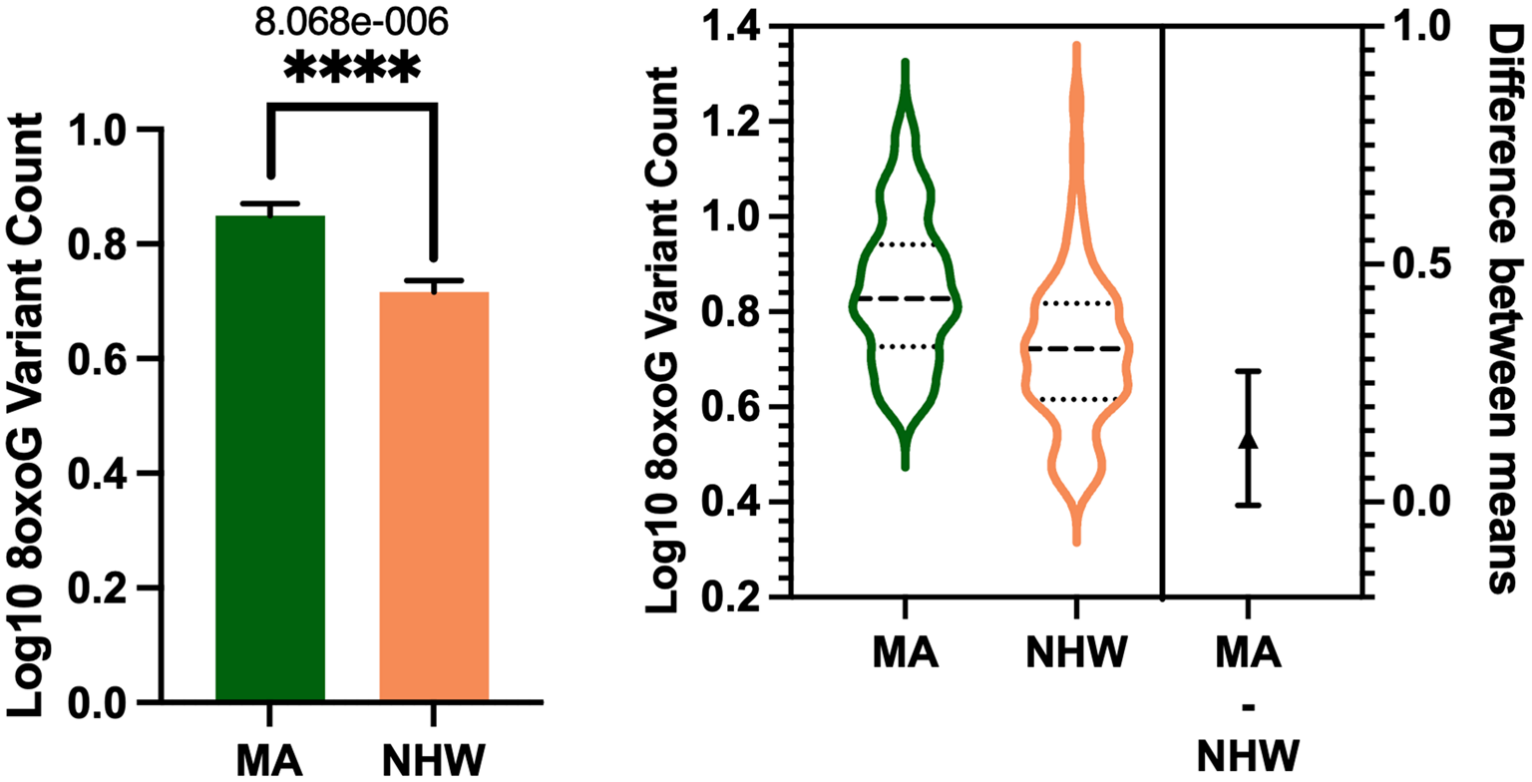
ccf-mtDNA 8oxoG variant count is significantly elevated in the Mexican American population. (**a**) Log transformed ccf-mtDNA 8oxoG variant count grouped by population using an unpaired, two-tailed t-test (*n* = 122, *t*-statistic = 4.666, df = 120, *P* ≤ 0.0001). Error bars represent standard error of the mean. (**b**) Violin plot demonstrating distribution of 8oxoG variant count in MAs and NHWs (n = 122) with effect size and confidence interval plotted on right y-axis. Dashed lines indicate the mean and dotted lines represent the 1st and 3^rd^ quartile. The triangle represents the difference of the means.

**Figure 2:**
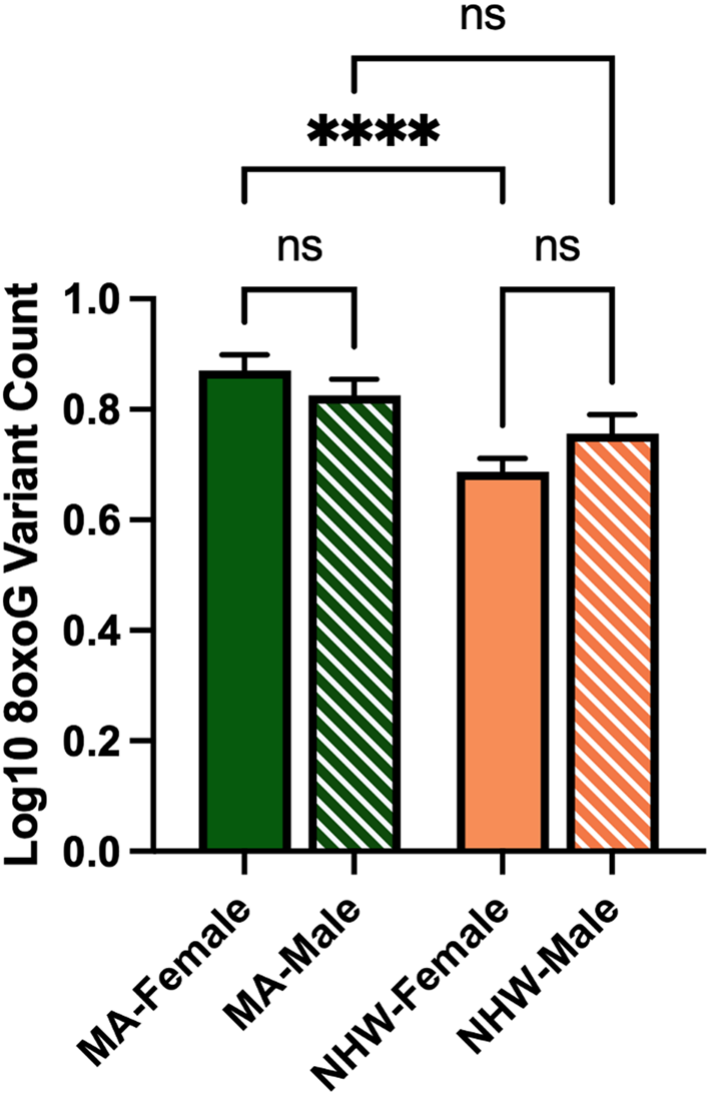
ccf-mtDNA 8oxoG variant count is significantly higher in Mexican American females compared to non-Hispanic White females. Log transformed plasma 8oxoG variant count by population and sex was analyzed using a two-way ANOVA (*n* = 122, *p* = 0.1275, F-statistic = 2.356, df = 118). Significant p-value for MA-Female 8oxoG variant count compared to NHW-Female (*p* = 2.515e-005). Error bars represent standard error of the mean.

**Figure 3:**
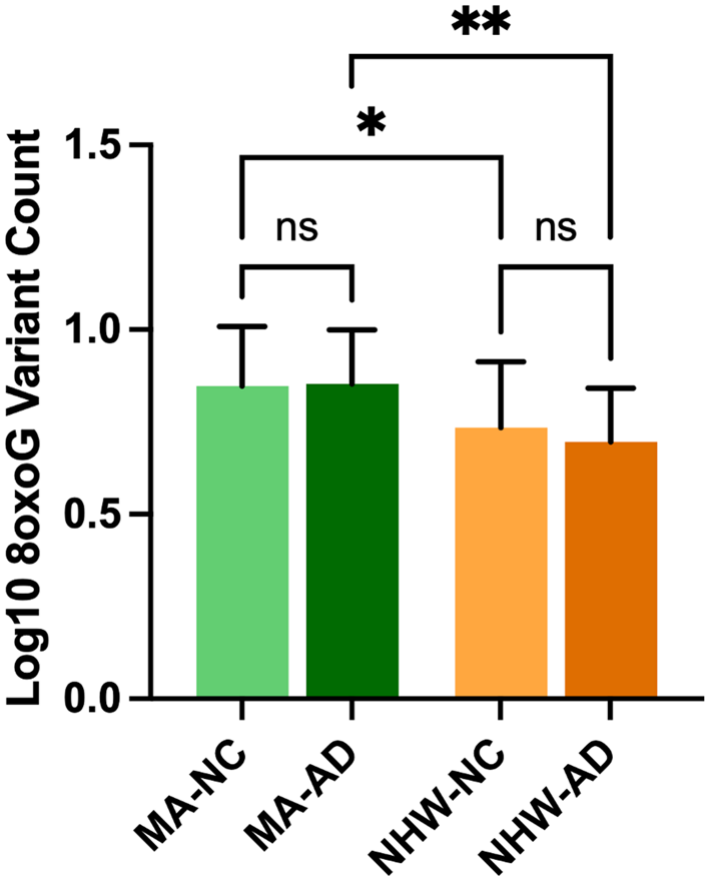
ccf-mtDNA 8oxoG variant count is significantly higher in Mexican Americans compared to non-Hispanic Whites despite cognitive phenotype. Log transformed plasma 8oxoG variant count by population and cognitive status was analyzed using a two-way ANOVA with interaction terms for population-cognition (*n* = 122, *P* = 0.4434, F-statistic = 0.5916, df = 118). There is no significant population × cognition interaction. Significant source of variation was caused by population (*P* ≤ 0.0001). Significant p-value for MA-NC 8oxoG variant count compared to NHW-NC (*P* = 0.0301) and MA-AD compared to NHW-AD (*P* = 0.0014). Error bars represent standard error of the mean.

**Figure 4:**
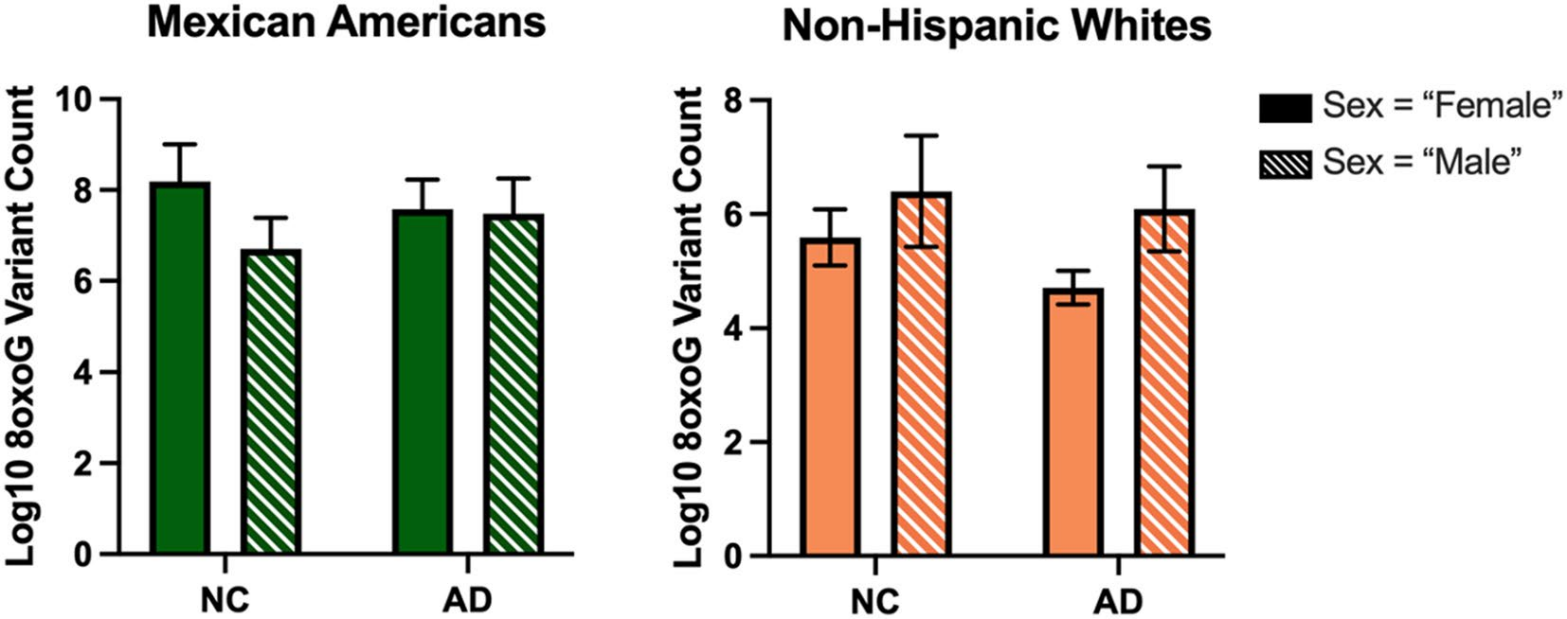
ccf-mtDNA 8oxoG variant count by cognitive phenotype and sex in each population. (**a**) Grouped bar graph of log transformed plasma 8oxoG variant count by cognition and sex in MAs testing for cognition × sex interaction via two-way ANOVA (*n* = 57, *P* = 0.3633, F-statistic = 0.8408, df = 53). (**b**) Grouped bar graph of log transformed plasma 8oxoG variant count by cognition and sex in NHWs testing for cognition × sex interaction via two-way ANOVA (*n* = 65, *P* = 0.6476, F-statistic = 0.2110, df = 61). Error bars represent standard error of the mean.

In the whole cohort ccf-8oxoG variant count was significantly elevated in the MA population compared to NHWs; mean = 0.8500 and 0.7160, respectively (Fig. 1). Ccf-8oxoG variant count did not significantly differ based on cognitive status or sex (Supplementary Figs. 4 and 5). A significant population × sex interaction was not observed (Fig. 2); however, ccf-8oxoG variant count was significantly elevated in MA females compared to NHW females (mean = 0.8702 and 0.7771, respectively). Despite cognitive phenotype, MAs had an elevated 8oxoG sSNV burden compared to NHWs (Fig. 3), yet there was not a significant difference for 8oxoG variant count in each population when assessing for sex and and cognitive phenotype (Fig. 4).

Results of multiple linear regression modelling of ccf-mtDNA 8oxoG variant count in the whole cohort with respect to population- sex and education interactive effects while considering APOE status as a dosage effect (i.e., no ε4 allele, one ε4 allele, two ε4 alleles) indicated a significant positive association for APOE and the population × sex interaction (Supplementary Table 23). When assessing for a diabetes × cognition interaction, a significant association between ccf-8oxoG variant count and both population and APOE was determined (Supplementary Table 24).

Additional multiple linear regression models were performed to characterize associations with 8oxoG “hotspots” in the whole cohort (Supplementary Fig. 6 and Supplementary Tables 25–26). The regression modelling for both interaction effects, population × sex and education, and diabetes × cognition in the whole cohort did not observe the same associations for 8oxoG “hotspots”, indicating these variants may not be informative in this context.

### Population-specific associations to ccf-8oxoG variant count

Population stratification for the multiple linear regression models did not demonstrate any statistical significance for ccf-8oxoG count in MAs (Table 5); however, in the NHW population AD and diabetes was marginally significant (Table 6). Further, the model showed significant statistical association with sex and age, as well.

**Table 5:** Multiple linear regression results for ccf-8oxoG variant count within Mexican Americans. Italics and bolding indicate a *P*-value of significance, while italics alone indicate a *P*-value approaching significance.

Variable	Coefficient	SE	t-statistic	*P*-value
Constant	0.9416425	0.3160556	2.979	***0.00495***
Cognitive Status with respect to AD	− 0.0213429	0.0538188	− 0.397	0.69385
Sex with respect to Male	− 0.0475924	0.0500437	− 0.951	0.34745
Age	− 0.0003705	0.0033248	− 0.111	0.91184
Years of Education	− 0.0005624	0.0052002	− 0.108	0.91443
BMI	− 0.0017609	0.005286	− 0.333	0.74082
Diabetes with respect to "Yes"	− 0.0280655	0.0557223	− 0.504	0.61733
Depression (with respect to "Yes")	− 0.0086758	0.0653148	− 0.133	0.89501
Tobacco Abuse (with respect to "Yes")	− 0.0399383	0.0532798	− 0.75	0.458
APOE	0.0629617	0.061671	1.021	0.31358
OGG1	0.0430802	0.0385428	1.118	0.27052
R-squared	0.1495		*P*-value	0.7312
Adjusted R-squared	− 0.06859		df	10 and 39
F-statistic	0.6855		Sample n	50

**Table 6:** Multiple linear regression results for ccf-8oxoG variant count within non-Hispanic Whites. Italics and bolding indicate a *P*-value of significance, while italics alone indicate a *P*-value approaching significance.

Variable	Coefficient	SE	t-statistic	*P*-value
Constant	1.115392	0.327981	3.401	***0.00182***
Cognitive Status with respect to AD	− 0.107136	0.05317	− 2.015	*0.05237*
Sex with respect to Male	0.107027	0.046919	2.281	***0.02934***
Age	− 0.007998	0.003727	− 2.146	***0.03954***
Years of Education	− 0.002487	0.008295	− 0.3	0.76624
BMI	0.009303	0.00555	1.676	0.10345
Diabetes with respect to "Yes"	− 0.096071	0.051379	− 1.87	*0.07068*
Depression (with respect to "Yes")	0.017801	0.048754	0.365	0.71742
Tobacco Abuse (with respect to "Yes")	− 0.06144	0.044716	− 1.374	0.17898
APOE	0.057165	0.039934	1.431	0.16199
OGG1	− 0.024474	0.036719	− 0.667	0.50986
R-squared	0.4417		*P*-value	0.2672
Adjusted R-squared	0.2672		df	10 and 32
F-statistic	2.532		Sample n	43

Multiple linear regression models for 8oxoG “hotspots” in each population were performed and no associations were observed in the MA population; however, in the NHW population a significant negative association to age was discerned (Supplementary Tables 27–28). Although the significant association to age in the NHW population was previously observed in the non- “hotspot” stratified regression model, generally the “hotspot” stratified analyses were less informative.

## Discussion

Ethnic/racial differences in developing cognitive impairment are known to exist, yet reports investigating biological, behavioral, and lifestyle factors that lead to differential mechanisms of neurodegeneration in populations more heavily burdened by cognitive decline are limited. Here, we investigated the predictability of 8oxoG variant count from two blood fractions in assessing risk for cognitive decline in two populations. We hypothesized that indirectly evaluating mitochondrial dysfunction through cellular mitochondrial 8oxoG sSNVs may serve as a better biomarker for MAs due to their observed metabolic endophenotype and burden, while ccf-mtDNA 8oxoG variants may provide improved utility as a biomarker for NHWs due to their inflammatory endophenotype.

Altogether, our results confirm MAs, especially females, show greater mtDNA oxidative damage compared to NHWs. Tobacco abuse trended for significance with increasing cellular 8oxoG mutational load in MAs; however, interestingly, non-depressed individuals showed elevated cellular 8oxoG variant burden. Stratified regression analysis by population in buffy coat PBMCs demonstrated an association with AD, sex, education, and tobacco abuse, while this was not observed in NHWs. Similarly, ccf-8oxoG variant load was significantly higher in MAs, and MA females had elevated levels compared to males. Ccf-8oxoG stratified regression analysis for NHWs showed a suggestive association with normal cognition in younger aged males without diabetes. This may indicate that oxidative variants from ccf-mtDNA are reduced in older NHW females with AD and diabetes comorbidity; however, the potential reason for this is unclear. There is growing evidence indicating the protective effects of estrogen against OS related damage^47,48^. Estrogen deficiency post menopause is associated with increased levels of OS, higher blood levels of free fatty acids, and reduced antioxidant defense^47,48^, so it is expected that aging females would demonstrate elevated levels of oxidative damage. Nonetheless, due to our observation it begs the question if there are sex-related differences for the age-related decline in mitophagy.

Depression is a known risk factor for developing MCI and AD, and a study demonstrated a depressive endophenotype of MCI and AD in MAs^49^. Furthermore, numerous studies report MAs experience more depressive symptoms compared to other Hispanic/Latino subpopulations^50,51^, as well as NHWs^52–57^. Accumulating evidence indicates that individuals with depression have higher levels of 8oxoG and oxidative damage, as OS encompasses a critical role in depression pathophysiology through the activity of ROS^58,59^. Substantial evidence indicates a higher prevalence of depression and depressive symptoms among MAs, yet our data denoted that non-depressed MAs exhibited elevated levels of 8oxoG variants in buffy coat PBMCs. However, depression appeared to be underrepresented because of limited reports of depression in MAs with MCI and AD. Subsequent regression models in the whole cohort and MA population show a negative trend between 8oxoG variant count in buffy PBMCs and non-depressed MAs. Surveying for the presence or absence of depression may have poor resolution when investigating cognitive associations compared to assessing for a collection of depressive symptoms. Previous studies report distinct clustering of depressive symptoms is imperative when studying the connection between cognition and depression^60,61^. Our results for 8oxoG and depression among MAs warrants further investigation by implementing depressive symptoms and/or other indicators of depression.

Our results revealed increased levels of 8oxoG variants in buffy PMBCs of MAs with a history of tobacco abuse. Smoking tobacco and exposure to tobacco smoke has been shown to cause elevated levels of 8oxoG compared to non-smokers^62–65^ because of the various carcinogens contained within^66,67^. As previously mentioned, carcinogens readily form DNA adducts and can lead to OS through the production of ROS. Additionally, cigarette smoke has been recognized to cause chronic inflammation leading to increasing OS which further results in accumulating oxidative damage^66,68^. A recent study reported that smoking tobacco increased risk for cognitive decline in aging MAs^69^. Following linear regression models including MAs all demonstrated a significant link with tobacco abuse. These results seem to indicate tobacco abuse as a strong modifiable risk factor for increased mitochondrial oxidative damage in MAs and demonstrates the importance of addressing such behaviors to prevent increased risk for CI in this population.

Our higher resolution linear regression models in the whole cohort from buffy coat PBMCs established that population (MA), tobacco abuse, BMI, education, and population × sex were statistically associated with 8oxoG somatic variants. It is well-established that social determinants of health can play a significant role in risk for disease, and the association between socioeconomic status and health outcome becomes more prominent with age^70^. When considering education as an associated factor in this context it is logical to question the influence of occupation and/or other socioeconomic factors (e.g., environmental exposure to pollutants) have on risk for cognitive impairment. There are numerous reports indicating that educational attainment is associated with healthy aging^70–74^, which may be attributed to educational opportunities influencing healthy behaviors, improved access to quality healthcare, better neighborhood quality, and employment outlook including careers with limited exposure to hazardous environments and/or strenuous labor, which can potentially translate to higher pay/income and life fulfillment^74^. Previous research has shown evidence that cognitive requirements from one’s predominant occupation is associated with age-related rate of cognitive decline (e.g., individuals that had lifetime careers that necessitated greater amounts of cognitive requirements had better cognitive function later in life)^75–78^. Within the framework that higher educational attainment leads to a better occupation and income which is likely to influence housing and surrounding environmental conditions, it would be expected that the inclusion of occupation and pollution would demonstrate significance as well in our linear regression models. Thus, occupation and pollution are likely important mediators in reducing the risk for cognitive impairment in the MA population similar to education, emphasizing racial inequities experienced by Mexican Americans as a result of structural racism. Structural racism contributes to environmental differences including but not limited to neighborhood and education quality, access to quality healthcare, pollutant and violence exposure, workforce opportunities and safety, and generational wealth^1,79–81^. Future studies investigating the relationship between cognitive decline and occupation, as well as pollution, in MAs may be crucial in demonstrating the necessity of programs that focus on mitigating the effects of structural racism.

AD was suggestively associated with cellular 8oxoG sSNVs when assessing an interaction between diabetes and cognitive status. These results were not observed in the NHW population, and perhaps of interest is the fact that many of the coefficients were in the opposite direction (though not significant). Increasing evidence connects AD and T2D, showing a greater risk for cognitive decline due to T2D, and robust correlations indicate high blood sugar is associated with the presence of Aβ plaques^82^. Brain dysfunction is frequently observed in earlier stages of T2D, and hemoglobin A1C (established biomarker for T2D) has been related to decline in functional memory and hippocampal size^82^. Links between T2D and AD implicate mitochondrial dysfunction as a participating factor in the development and/or progression of neurodegeneration and may be of exceptional importance for ethnic/racial differences in disease severity and manifestation. This evidence further suggests that mitochondrial health could be a contributor to the unexplained disparities in CI among MAs, especially since MAs are at great risk for metabolic disorders.

It is important to note that this study has its limitations including (1) measuring 8oxoG lesions indirectly, (2) small sampling for the plasma dataset, (3) lacking biochemical, metabolic, and inflammatory phenotypes, (4) missing most nDNA variants, (5) solely sampling blood tissue, (6) evaluating in one cohort, and (7) APOE in the NHW model could have reduced power due to a larger number of missingness compared to MAs. Additionally, another limitation that could be considered is the absence of cell composition data to adjust for differences in mitochondrial content that may introduce variability in variant allele frequency among both cell and ethnic/racial populations. To better understand the biological and mechanistic roles of mitochondrial dysfunction and oxidative DNA damage in this context, future studies should incorporate larger sampling, include more biological markers indicative of metabolic health and systemic inflammation, determine if utilizing MMSE, CDR sum, and/or other neuropsychological tests for cognitive function strengthens our power to further support/validate our results, and characterize the nuclear genetic background associated with the mtDNA for each subject. Future studies should also investigate the mechanistic role of mitochondrial processes, such as mitophagy and mitochondrial quality control and sensing, play in contributing to the observed differences in cellular and ccf-mtDNA 8oxoG sSNVs between MAs and NHWs in relation to cognitive decline. Additionally, future studies will aim to validate the applicability of peripheral cellular and cell-free pathophysiological phenotypes as biomarkers for assessing brain pathology, disease risk, and/or disease stage. These studies will also investigate expression of DNA repair machinery, the role of sex hormones, and validate oxidative mtDNA load using alternative methods.

**Figure 5:**
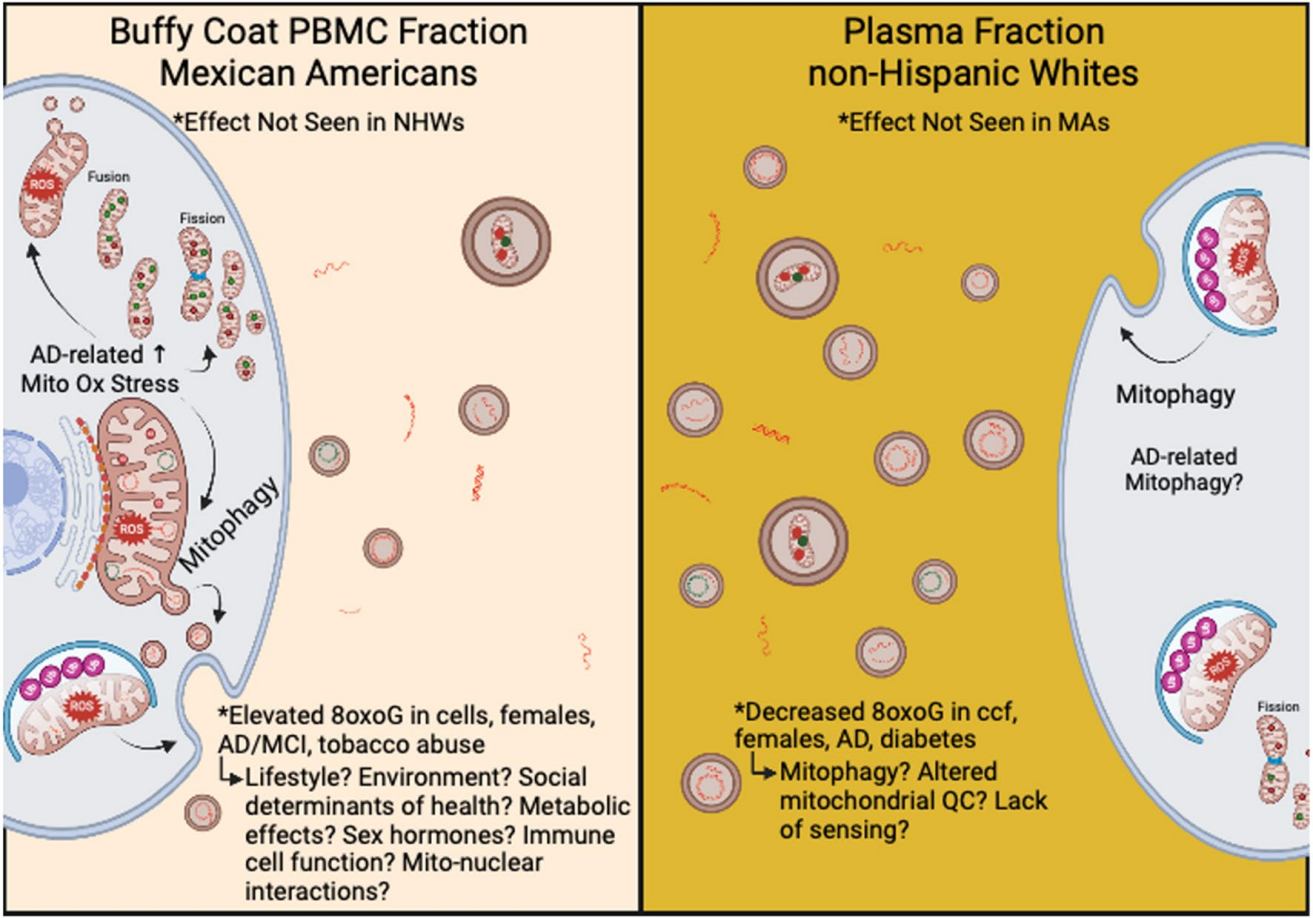
Hypothetical schematic representing the potential mechanism(s) observed from cellular and ccf-mtDNA 8oxoG variants in MA vs NHW participants of TARCC. In the buffy coat PBMC blood fraction (left) observations in cellular 8oxoG variant count were found to be significant in MAs and the effects were not observed in NHWs. In buffy coat PBMCs, cellular 8oxoG variant count was significantly elevated in MAs, especially females, and was associated with CI and tobacco abuse. Modifiable risk factors, biological processes, and genetics such as lifestyle, environment, social determinants of health, immune cell function, and mito-nuclear interactions are theorized to contribute to elevated oxidative damage to mtDNA in MAs from TARCC. In the blood plasma fraction (right) observations of ccf-8oxoG variant count were significantly reduced in NHW females and were associated with AD and diabetes. These results suggest possible alterations in mitochondrial quality control and/or lack of sensing in NHW females. Overall results from cellular and ccf-mtDNA 8oxoG variant loads suggest mitophagy may play a role in ethnic/racial differences in AD etiology. This figure was created with BioRender.com.

Uniquely, our data specifically point to novel, population-based effects in 8oxoG damage in cellular and cell-free mtDNA. Overall, our results indicate both cellular and ccf-8oxoG variants mtDNA are significantly elevated in MAs compared to NHWs and indicate sex-differences (Fig. 5). Notably, our results from evaluating oxidized cellular mtDNA compared to ccf-mtDNA presents the possibility that these biomarkers may have significant predictive capability in MAs, especially females, compared to NHWs due to the observed statistical significance of assessing various independent variables. This evidence implies mitochondrial dysfunction in cellular mtDNA may be distinctly related to disease pathology in MAs with cognitive decline, whereas in ccf-mtDNA displayed poor associations in MAs compared to NHWs. This cumulative evidence supports the notion that (1) blood-based signatures of mitochondrial dysfunction differ between ethnic/racial populations, (2) cellular and ccf-mtDNA possess different functionality in potentially developing pathophysiological conditions, and (3) ethnic/racial differences exist in the manifestation of neurodegeneration through the assessment of mitochondrial oxidative DNA damage from different blood fractions.

## Methods

### Sample acquisition and description

#### Cohort

The Texas Alzheimer’s Research and Care Consortium (TARCC) is a population-based collaborative longitudinal research initiative that has expanded between several Texas medical research institutions^83^. TARCC explores factors that may attribute to the development and progression of cognitive impairment due to AD in the MA population compared to their NHW counterparts.

#### Participants

The study received institutional review board approval under the University of North Texas Health Science Center IRB #1330309-1 and all experiments were performed in accordance with relevant guidelines and regulations. Informed written consent was obtained from participants and/or their legally authorized proxies to take part in the study and allow publication of findings before data collection. Volunteer aging participants enrolled in TARCC annually complete a medical evaluation, clinical interview, neuropsychological testing, and blood draw. Eligible participants obtained categorical clinical diagnoses of ‘Alzheimer’s disease’, ‘Mild Cognitive Impairment’, and ‘Normal Control’ based on the criteria provided by the National Institute for Neurological Communicative Disorders and Stroke-Alzheimer’s Disease and Related Disorders Association^84^. Additional information regarding the inclusion and exclusionary criteria of TARCC has been discussed elsewhere^49^. This study included NHW and MA subjects (N = 559; Table 1) diagnosed with Alzheimer’s Disease (n = 104), Mild Cognitive Impairment (n = 127), or normal cognition (n = 328). Obtained buffy coat samples from NHWs (n = 261) and MAs (n = 299) and a subset of plasma samples from 62 NHWs and 57 MAs (N = 119; Table 4) collected at the same visit were selected to match the distribution of subjects with respect to age, sex, and type-2 diabetes among both populations. The plasma subset did not include individuals diagnosed with MCI. These samples were analyzed to characterize cellular and circulating cell-free mtDNA (ccf-mtDNA) oxidative damage from blood.

### Measurement of mtDNA mutational load from buffy coat and plasma

#### DNA extraction

DNA from both the buffy coat and plasma was extracted individually from 200 μL of each sample using the Mag-Bind^®^ Blood & Tissue DNA HDQ 96 kit (Omega Bio-tek, Norcross, GA). Buffy coat and plasma DNA extractions were conducted using the Hamilton Microlab STARlet automated liquid handler (Hamilton Company, Reno, NV) and manually, respectively.

#### Whole mtDNA amplification

The whole mitochondrial genome and large mtDNA fragments for each sample were amplified using the REPLI-g^®^ Human Mitochondrial DNA kit (Qiagen, Venlo, Netherlands) following the manufacturer’s protocol. This kit uses the high fidelity proofreading phi29 DNA polymerase capable of both rolling circle and multiple displacement amplification in combination of random hexamers^85^. Mitochondrial genome amplification was performed in order to increase mtDNA levels relative to nuclear DNA to enhance mtDNA coverage for whole genome sequencing. Amplified product was quantified via Qubit dsDNA BR assay on the Qubit 4 fluorometer (Invitrogen^TM^, Thermo Fisher Scientific, Waltham, MA) for each sample and a small test size of approximately 12 samples were evaluated to determine the distribution of amplicon sizes using the 4200 TapeStation System (Agilent Technologies, Santa Clara, CA) following the manufacturer’s protocol. The Genomic DNA ScreenTape and corresponding reagents were used to determine the presence of mtDNA fragments from 200 bp to the whole genome.

#### mtDNA sequencing

The Nextera XT^TM^ DNA Library Preparation kit (Illumina, San Diego, CA) was used to prepare the sample library for sequencing following the manufacturer’s protocol. All samples were sequenced on the NextSeq 550 Sequencer (Illumina) platform with high output kit v2.5 generating paired-end reads of 150 bp for 300 cycles. The buffy coat samples had an average read depth of 1855X, while the plasma samples had an average read depth of 3970X.

#### mtDNA sequence mapping/alignment and variant calling

The methods section of Reid, et al., outlines sequence mapping/alignment and mtDNA variant identification for the processing of raw mtDNA gzipped FASTQ pairs generated for each sample^24^.

#### Identification of biological variants indicative of oxidative damage

Considering possible bias from the potential presence of basecalls derived from technical oxidative damage as explained in Reid, et al., basecalls were evaluated and manually reviewed as detailed in Reid, et al.^24^. Variants indicative of 8oxoG damage for each subject from the buffy coat portion were summed and normalized by accounting for read depth (variant count per 1000 read depth) to evaluate group differences based on the following variables: population, cognition, sex, type-2 diabetes, comorbidity (cognitive impairment and diabetes), both *APOE* and *OGG1* genotype, and lifestyle factors. Variants indicative of 8oxoG damage from plasma was summed and normalized by accounting for read depth and were log10 transformed to evaluate group differences with the same variables described above. 8oxoG variant “hotspots” were identified as previously indicated in Reid et al.^24^.

#### APOE and OGG1 genotyping imputation

Genome-wide SNP profiles were generated using the Illumina Infinium Multi-Ethnic Global Array which types 1.7million SNPs. Standard filtering based on SNP missingness, individual missingness, and minor allele frequency (5%) was conducted according to Anderson et al., 2010^86^. Genetic imputation of APOE (rs7412 and rs429358 for individuals missing APOE genotypes) and OGG1 (rs1052133) was performed using Impute2 based on the 1000 Genomes Project Phase 3 data; probabilistic genotypes for were called at a threshold of 0.8.

#### Statistical analyses

Statistical analyses were performed using Microsoft Excel, IBM SPSS software (v. 27.0), R software (v. 4.2.0), and GraphPad Prism software (v. 9.4.0). Welch’s t-test (two-tailed) and two-way ANOVA were performed on 8oxoG mutational load to compare between both population groups. Multiple linear regression analysis was performed to evaluate the relationship between cognition, sex, age, education, diabetes and depression status, and tobacco abuse with 8oxoG variant count both within the whole study cohort and in stratified analyses of MAs and NHWs.

### Supplementary Information

Supplementary Information.

## Data Availability

The data that support the findings of this study are subject to restrictions that limit their public availability; permission was obtained for use of these data under the current study. Data are available in a Synapse data repository from the corresponding author Nicole Phillips upon reasonable request.
