# Supplementary material for: Integrative blood-based characterization of oxidative mitochondrial DNA damage variants implicates Mexican American’s metabolic risk for developing Alzheimer’s disease
Source: Sci Rep. 2023 Sep 7;13:14765. doi: 10.1038/s41598-023-41190-6 (PMC10484983; doi:10.1038/s41598-023-41190-6)
Supplement: Supplementary file 1 — Supplementary Information. [file 41598_2023_41190_MOESM1_ESM.docx]

**Assessment of cellular mtDNA 8oxoG Variant Count in MA and NHW TARCC Participants**

**Supplementary Table 1.** *APOE* genotype directly sequenced vs. imputed in buffy coat of TARCC participants by population.

| **Non-Hispanic Whites** | **N = 260** |
| --- | --- |
| *APOE* Genotyped | 216 (83.1%) |
| *APOE* Imputed | 22 (8.5%) |
| *APOE* Missing | 22 (8.5%) |
| **Mexican Americans** | **N = 299** |
| *APOE* Genotyped | 294 (98.3%) |
| *APOE* Imputed | 4 (1.3%) |
| *APOE* Missing | 1 (0.33%) |

**Supplementary Table 2.** *APOE* and *OGG1* genotype frequencies in each population based on cognitive phenotype in TARCC participants assessed via buffy coat.

| **Non-Hispanic Whites** | | **NC** | **MCI** | **AD** |
| --- | --- | --- | --- | --- |
| *APOE* Genotype  (n = 238) | ε2/ε2 | 1 (0.65%) | 0 (0%) | 0 (0%) |
|  | ε2/ε3 | 15 (9.8%) | 6 (14%) | 1 (1.6%) |
|  | ε2/ε4 | 3 (1.96%) | 0 (0%) | 0 (0%) |
|  | ε3/ε3 | 85 (55.6%) | 21 (48.8%) | 19 (29.7%) |
|  | ε3/ε4 | 24 (15.7%) | 9 (20.9%) | 30 (46.9%) |
|  | ε4/ε4 | 4 (2.6%) | 7 (16.3%) | 13 (20.3%) |
|  |  |  |  |  |
| *OGG1* Genotype  (n = 184) | Ser326 | 53 (34.6%) | 21 (48.8%) | 30 (46.9%) |
|  | Ser/Cys326 | 47 (30.7%) | 8 (18.6%) | 16 (25%) |
|  | Cys326 | 5 (3.3%) | 1 (2.3%) | 3 (4.7%) |
|  |  |  |  |  |
| **Mexican Americans** | | **NC** | **MCI** | **AD** |
| *APOE* Genotype  (n = 298) | ε2/ε2 | 1 (0.57%) | 0 (0%) | 0 (0%) |
|  | ε2/ε3 | 17 (9.7%) | 3 (3.6%) | 0 (0%) |
|  | ε2/ε4 | 0 (0%) | 0 (0%) | 0 (0%) |
|  | ε3/ε3 | 121 (69.1%) | 61 (72.6%) | 25 (62.5%) |
|  | ε3/ε4 | 33 (18.9%) | 18 (21.4%) | 15 (37.5%) |
|  | ε4/ε4 | 2 (1.1%) | 2 (2.3%) | 0 (0%) |
|  |  |  |  |  |
| *OGG1* Genotype  (n = 242) | Ser326 | 55 (31.4%) | 28 (33.3%) | 18 (45%) |
|  | Ser/Cys326 | 68 (38.9%) | 31 (36.9%) | 13 (32.5%) |
|  | Cys326 | 17 (9.7%) | 8 (9.5%) | 4 (10%) |

**Supplementary Table 3.** Hardy-Weinberg equilibrium check for *APOE* in TARCC participants.

|  | **Total Cohort (N = 536)** | **Non-Hispanic Whites (N = 238)** | **Mexican Americans (N = 298)** |
| --- | --- | --- | --- |
| ε2 | 0.04571 | 0.05672 | 0.03691 |
| ε3 | 0.7789 | 0.7038 | 0.8389 |
| ε4 | 0.1753 | 0.2395 | 0.1242 |
| Observed ε2/ε2 | 0.003731 | 0.004202 | 0.003356 |
| Observed ε2/ε3 | 0.07836 | 0.09244 | 0.06711 |
| Observed ε2/ε4 | 0.005597 | 0.01261 | 0.00 |
| Observed ε3/ε3 | 0.6194 | 0.5252 | 0.6946 |
| Observed ε3/ε4 | 0.2407 | 0.2647 | 0.2215 |
| Observed ε4/ε4 | 0.05224 | 0.1008 | 0.01342 |
| Expected ε2/ε2 | 0.002089 | 0.003217 | 0.001363 |
| Expected ε2/ε3 | 0.07121 | 0.07984 | 0.06193 |
| Expected ε2/ε4 | 0.01603 | 0.02717 | 0.009166 |
| Expected ε3/ε3 | 0.6067 | 0.4953 | 0.7038 |
| Expected ε3/ε4 | 0.2732 | 0.3371 | 0.2083 |
| Expected ε4/ε4 | 0.03076 | 0.05736 | 0.01542 |
| ***p*-value** | ***0.9861*** | ***0.9702*** | ***0.9932*** |

**Supplementary Table 4.** Hardy-Weinberg equilibrium check for *OGG1* in TARCC participants.

|  | **Total Cohort (N = 426)** | **Non-Hispanic Whites (N = 184)** | **Mexican Americans (N = 242)** |
| --- | --- | --- | --- |
| Ser326 | 0.6960 | 0.7582 | 0.6488 |
| Cys326 | 0.3040 | 0.2418 | 0.3512 |
| Observed Ser326/Ser326 | 0.4812 | 0.5652 | 0.4174 |
| Observed Ser326/Cys326 | 0.4296 | 0.3859 | 0.4628 |
| Observed Cys326/Cys326 | 0.08920 | 0.04891 | 0.1198 |
| Expected Ser326/Ser326 | 0.4844 | 0.5748 | 0.4209 |
| Expected Ser326/Cys326 | 0.4232 | 0.3667 | 0.4557 |
| Expected Cys326/Cys326 | 0.09241 | 0.05849 | 0.1234 |
| ***p*-value** | ***0.9879*** | ***0.9583*** | ***0.9876*** |

**Supplementary Figure 1. Cellular 8oxoG variant count is significantly higher in self-reported non-depressed Mexican Americans. a** Total 8oxoG variant count was assessed by depression status using a two-tailed Welch’s t-test (n = 299, t-statistic = 2.010, df = 105.8, p = 0.04693). Error bars represent standard error of the mean. **b** Violin plot displaying the distribution of 8oxoG variant counts in individuals with and without depression (n = 559) with effect size and confidence interval plotted on right y-axis. Dashed lines indicate the mean and dotted lines represent the 1st and 3rd quartile. The triangle represents the difference of the means, and the associated bar indicates the confidence interval.

**Supplementary Figure 2. Total Cellular 8oxoG count approaches significance in MA individuals with a history of tobacco abuse. a** Total 8oxoG variant count was evaluated by history of tobacco abuse using a two-tailed Welch’s t-test (n = 299, t-statistic = 1.751, df = 257.2, *p* = 0.0812). Error bars represent standard error of the mean. **b** Violin plot displaying the distribution of 8oxoG variant counts in individuals with and without a history of tobacco abuse (n = 559) with effect size and confidence interval plotted on right y-axis. Dashed lines indicate the mean and dotted lines represent the 1st and 3rd quartile. The triangle represents the difference of the means, and the associated bar indicates the confidence interval.

**Supplementary Table 5.** Cellular 8oxoG variant count and cognitive status (NC vs MCI or AD) multiple linear regression model prediction considering population interaction effect with both sex and education. Italics and bolding indicate a p-value of significance, while italics alone indicate a p-value approaching significance.

| **Variable** | **Coefficient** | **Std. Error** | **t-statistic** | ***p*-value** |
| --- | --- | --- | --- | --- |
| Constant | 3.12218 | 2.50099 | 1.248 | 0.212613 |
| Population (with respect to NHW) | -1.74971 | 1.79942 | -0.972 | 0.331444 |
| Cognitive Status (with respect to AD) | 0.72882 | 0.54067 | 1.348 | 0.178412 |
| Cognitive Status (with respect to MCI) | 0.59658 | 0.49557 | 1.204 | 0.229364 |
| Sex (with respect to Male) | -1.79283 | 0.52766 | -3.398 | ***0.000747*** |
| Age | 0.01725 | 0.02955 | 0.584 | 0.559639 |
| Years of Education | 0.15024 | 0.05377 | 2.794 | ***0.005456*** |
| BMI | 0.0797 | 0.03633 | 2.193 | ***0.028841*** |
| Diabetes (with respect to "Yes") | -0.63683 | 0.42547 | -1.497 | 0.135232 |
| Depression (with respect to "Yes") | -0.72191 | 0.52278 | -1.381 | 0.168071 |
| Tobacco Abuse (with respect to "Yes") | 1.04508 | 0.39607 | 2.639 | ***0.008646*** |
| APOE | 0.16904 | 0.35467 | 0.477 | 0.63389 |
| OGG1 | -0.12608 | 0.29831 | -0.423 | 0.672781 |
| Interaction: NHW x Male "Yes" | 2.26289 | 0.77646 | 2.914 | ***0.003762*** |
| Interaction: NHW x Years of Education | -0.11933 | 0.11921 | -1.001 | 0.31739 |

| R-squared | 0.1221 | *p*-value | 1.565e-06 |
| --- | --- | --- | --- |
| Adjusted R-squared | 0.09172 | df | 14 and 405 |
| F-statistic | 4.022 | Sample n | 420 |

**Supplementary Table 6.** Cellular 8oxoG variant count and cognitive status (NC vs MCI or AD) multiple linear regression model prediction considering diabetes interaction effect with cognitive status. Italics and bolding indicate a p-value of significance, while italics alone indicate a p-value approaching significance.

| **Variable** | **Coefficient** | **Std. Error** | **t-statistic** | ***p*-value** |
| --- | --- | --- | --- | --- |
| Constant | 2.16016 | 2.50269 | 0.863 | 0.3886 |
| Population (with respect to NHW) | -2.35566 | 0.4857 | -4.85 | ***1.76e-06*** |
| Cognitive Status (with respect to AD) | 1.24926 | 0.65088 | 1.919 | *0.0556* |
| Cognitive Status (with respect to MCI) | 0.54274 | 0.62565 | 0.867 | 0.3862 |
| Sex (with respect to Male) | -0.81169 | 0.39958 | -2.031 | ***0.0429*** |
| Age | 0.02659 | 0.0297 | 0.895 | 0.3711 |
| Years of Education | 0.12472 | 0.04877 | 2.557 | ***0.0109*** |
| BMI | 0.08239 | 0.03673 | 2.243 | ***0.0254*** |
| Diabetes (with respect to "Yes") | -0.24581 | 0.56673 | -0.434 | 0.6647 |
| Depression (with respect to "Yes") | -0.62874 | 0.52599 | -1.195 | 0.2326 |
| Tobacco Abuse (with respect to "Yes") | 0.96754 | 0.3979 | 2.432 | ***0.0155*** |
| APOE | 0.15846 | 0.35808 | 0.443 | 0.6584 |
| OGG1 | -0.20177 | 0.3006 | -0.671 | 0.5025 |
| Interaction: AD x Diabetes "Yes" | -1.38959 | 1.02584 | -1.355 | 0.1763 |
| Interaction: MCI x Diabetes "Yes" | -0.06118 | 0.98237 | -0.062 | 0.9504 |

| R-squared | 0.107 | *p*-value | 2.31e-05 |
| --- | --- | --- | --- |
| Adjusted R-squared | 0.07615 | df | 14 and 405 |
| F-statistic | 3.467 | Sample n | 420 |

**Supplementary Table 7.** Cellular 8oxoG “hotspot” variant count and cognitive status (NC vs MCI or AD) multiple linear regression model prediction considering *OGG1* genotype and population interaction effect with both sex and education. Italics and bolding indicate a p-value of significance, while italics alone indicate a p-value approaching significance.

| **Variable** | **Coefficient** | **Std. Error** | **t-statistic** | ***p*-value** |
| --- | --- | --- | --- | --- |
| Constant | 2.262487 | 1.248584 | 1.812 | ***0.0707*** |
| Population (with respect to NHW) | -1.267441 | 0.898335 | -1.411 | 0.159 |
| Cognitive Status (with respect to AD) | -0.051627 | 0.26992 | -0.191 | 0.8484 |
| Cognitive Status (with respect to MCI) | -0.038292 | 0.247408 | -0.155 | 0.8771 |
| Sex (with respect to Male) | -0.213908 | 0.263429 | -0.812 | 0.4173 |
| Age | 0.004708 | 0.014752 | 0.319 | 0.7498 |
| Years of Education | 0.007887 | 0.026846 | 0.294 | 0.7691 |
| BMI | 0.025208 | 0.018139 | 1.39 | 0.1654 |
| Diabetes (with respect to "Yes") | -0.160855 | 0.212408 | -0.757 | 0.4493 |
| Depression (with respect to "Yes") | -0.096772 | 0.260993 | -0.371 | 0.711 |
| Tobacco Abuse (with respect to "Yes") | 0.205513 | 0.197733 | 1.039 | 0.2993 |
| APOE | 0.086777 | 0.177063 | 0.49 | 0.6243 |
| OGG1 | -0.154641 | 0.148929 | -1.038 | 0.2997 |
| Interaction: NHW x Male "Yes" | 0.41861 | 0.387636 | 1.08 | 0.2808 |
| Interaction: NHW x Years of Education | 0.044539 | 0.059512 | 0.748 | 0.4547 |

| R-squared | 0.2393 | *p*-value | 0.7656 |
| --- | --- | --- | --- |
| Adjusted R-squared | -0.009812 | df | 14 and 405 |
| F-statistic | 0.7092 | Sample n | 420 |

**Supplementary Table 8.** Cellular 8oxoG “hotspot” variant count and cognitive status (NC vs MCI or AD) multiple linear regression model prediction considering *OGG1* genotype as well as diabetes and cognitive status interaction. Italics and bolding indicate a p-value of significance, while italics alone indicate a p-value approaching significance.

| **Variable** | **Coefficient** | **Std. Error** | **t-statistic** | ***p*-value** |
| --- | --- | --- | --- | --- |
| Constant | 2.036356 | 1.236478 | 1.647 | 0.1004 |
| Population (with respect to NHW) | -0.37884 | 0.239963 | -1.579 | 0.1152 |
| Cognitive Status (with respect to AD) | -0.06664 | 0.321575 | -0.207 | 0.8359 |
| Cognitive Status (with respect to MCI) | 0.266971 | 0.309107 | 0.864 | 0.3883 |
| Sex (with respect to Male) | 0.003812 | 0.197416 | 0.019 | ***9.85e-01*** |
| Age | 0.005012 | 0.014672 | 0.342 | 0.7328 |
| Years of Education | 0.015568 | 0.024098 | 0.646 | 0.5186 |
| BMI | 0.023945 | 0.018147 | 1.319 | 0.1878 |
| Diabetes (with respect to "Yes") | 0.053767 | 0.279997 | 0.192 | 0.8478 |
| Depression (with respect to "Yes") | -0.10751 | 0.259868 | -0.414 | 0.6793 |
| Tobacco Abuse (with respect to "Yes") | 0.161687 | 0.196587 | 0.822 | 0.4113 |
| APOE | 0.061665 | 0.176915 | 0.349 | 0.7276 |
| OGG1 | -0.13653 | 0.148512 | -0.919 | 0.3585 |
| Interaction: AD x Diabetes "Yes" | 0.044303 | 0.506826 | 0.087 | 0.9304 |
| Interaction: MCI x Diabetes "Yes" | -0.86358 | 0.48535 | -1.779 | ***0.0759*** |

| R-squared | 0.02768 | *p*-value | 0.6436 |
| --- | --- | --- | --- |
| Adjusted R-squared | -0.005934 | df | 14 and 405 |
| F-statistic | 0.8235 | Sample n | 420 |

**Supplementary Table 9.** Cellular 8oxoG “hotspot” variant count and cognitive status (NC vs MCI or AD) multiple linear regression model prediction considering *OGG1* genotype within the Mexican American population. Italics and bolding indicate a p-value of significance, while italics alone indicate a p-value approaching significance.

| **Variable** | **Coefficient** | **Std. Error** | **t-statistic** | ***p*-value** |
| --- | --- | --- | --- | --- |
| Constant | 3.186749 | 1.725239 | 1.847 | *0.066* |
| Cognitive Status (with respect to AD) | 0.082055 | 0.425397 | 0.193 | 0.847 |
| Cognitive Status (with respect to MCI) | -0.086177 | 0.337315 | -0.255 | 0.799 |
| Sex (with respect to Male) | -0.329255 | 0.292614 | -1.125 | 0.262 |
| Age | -0.004077 | 0.020858 | -0.195 | 0.845 |
| Years of Education | 0.003534 | 0.02952 | 0.12 | 0.905 |
| BMI | 0.014829 | 0.023773 | 0.624 | 0.533 |
| Diabetes (with respect to "Yes") | -0.254014 | 0.292223 | -0.869 | 0.386 |
| Depression (with respect to "Yes") | -0.375336 | 0.375911 | -0.998 | 0.319 |
| Tobacco Abuse (with respect to "Yes") | 0.454095 | 0.283764 | 1.6 | 0.111 |
| APOE | 0.206517 | 0.307277 | 0.672 | 0.502 |
| OGG1 | -0.088752 | 0.201158 | -0.441 | 0.659 |

| R-squared | 0.02522 | *p*-value | 0.8775 |
| --- | --- | --- | --- |
| Adjusted R-squared | -0.02181 | df | 11 and 228 |
| F-statistic | 0.5363 | Sample n | 201 |

**Supplementary Table 10.** Cellular 8oxoG “hotspot” variant count and cognitive status (NC vs MCI or AD) multiple linear regression model prediction considering *OGG1* genotype within the non-Hispanic White population. Italics and bolding indicate a p-value of significance, while italics alone indicate a p-value approaching significance.

| **Variable** | **Coefficient** | **Std. Error** | **t-statistic** | ***p*-value** |
| --- | --- | --- | --- | --- |
| Constant | 0.240313 | 1.997209 | 0.12 | 0.904 |
| Cognitive Status (with respect to AD) | -0.053166 | 0.33765 | -0.157 | 0.875 |
| Cognitive Status (with respect to MCI) | 0.276141 | 0.382296 | 0.722 | 0.471 |
| Sex (with respect to Male) | 0.188589 | 0.267741 | 0.704 | 0.482 |
| Age | 0.009629 | 0.021702 | 0.444 | 0.658 |
| Years of Education | 0.06132 | 0.049302 | 1.244 | 0.215 |
| BMI | 0.040103 | 0.029228 | 1.372 | 0.172 |
| Diabetes (with respect to "Yes") | -0.097021 | 0.313935 | -0.309 | 0.758 |
| Depression (with respect to "Yes") | 0.35329 | 0.381463 | 0.926 | 0.356 |
| Tobacco Abuse (with respect to "Yes") | -0.119536 | 0.272527 | -0.439 | 0.661 |
| APOE | -0.079565 | 0.215729 | -0.369 | 0.713 |
| OGG1 | -0.287485 | 0.223417 | -1.287 | 0.2 |

| R-squared | 0.04725 | *p*-value | 0.6819 |
| --- | --- | --- | --- |
| Adjusted R-squared | -0.01513 | df | 11 and 168 |
| F-statistic | 0.7574 | Sample n | 180 |

**Supplementary Table 11.** Total cellular 8oxoG variant count multiple linear regression model prediction considering cognitive impairment (NC vs MCI + AD), *OGG1* genotype, and population interaction effect with both sex and years of education. Italics and bolding indicate a p-value of significance, while italics alone indicate a p-value approaching significance.

| **Variable** | **Coefficient** | **Std. Error** | **t-statistic** | ***p*-value** |
| --- | --- | --- | --- | --- |
| Constant | 3.09706 | 2.4955 | 1.241 | 0.215301 |
| Population (with respect to NHW) | -1.74819 | 1.7973 | -0.973 | 0.331292 |
| Cognitive Impairment | 0.65399 | 0.42223 | 1.549 | 0.122185 |
| Sex (with respect to Male) | -1.80365 | 0.52479 | -3.437 | ***0.000649*** |
| Age | 0.01777 | 0.02942 | 0.604 | 0.546137 |
| Years of Education | 0.15093 | 0.05362 | 2.815 | ***0.005119*** |
| BMI | 0.0789 | 0.03611 | 2.185 | ***0.029476*** |
| Diabetes (with respect to "Yes") | -0.63093 | 0.42414 | -1.488 | 0.137643 |
| Depression (with respect to "Yes") | -0.71806 | 0.52188 | -1.376 | 0.169612 |
| Tobacco Abuse (with respect to "Yes") | 1.04785 | 0.39541 | 2.65 | ***0.008363*** |
| APOE | 0.18073 | 0.35033 | 0.516 | 0.606218 |
| OGG1 | -0.12746 | 0.2979 | -0.428 | 0.668967 |
| Interaction: NHW x Male "Yes" | 2.27335 | 0.77412 | 2.937 | ***0.003506*** |
| Interaction: NHW x Years of Education | -0.11929 | 0.11907 | -1.002 | 0.316985 |

| R-squared | 0.122 | *p*-value | 7.353e-07 |
| --- | --- | --- | --- |
| Adjusted R-squared | 0.09385 | df | 13 and 406 |
| F-statistic | 4.338 | Sample n | 420 |

**Supplementary Table 12.** Total cellular 8oxoG variant count multiple linear regression model prediction considering cognitive impairment (NC vs MCI + AD), *OGG1* genotype, and diabetes interaction effect. Italics and bolding indicate a p-value of significance, while italics alone indicate a p-value approaching significance.

| **Variable** | **Coefficient** | **Std. Error** | **t-statistic** | ***p*-value** |
| --- | --- | --- | --- | --- |
| Constant | 2.20767 | 2.4953 | 0.885 | 0.3768 |
| Population (with respect to NHW) | -2.31379 | 0.48336 | -4.787 | ***2.37e-06*** |
| Cognitive Impairment | 0.8922 | 0.51652 | 1.727 | *0.0849* |
| Sex (with respect to Male) | -0.79321 | 0.39745 | -1.996 | ***0.0466*** |
| Age | 0.02638 | 0.02955 | 0.893 | 0.3725 |
| Years of Education | 0.12382 | 0.0486 | 2.548 | ***0.0112*** |
| BMI | 0.08066 | 0.03652 | 2.208 | ***0.0278*** |
| Diabetes (with respect to "Yes") | -0.22149 | 0.56487 | -0.392 | 0.6952 |
| Depression (with respect to "Yes") | -0.69608 | 0.5208 | -1.337 | 0.1821 |
| Tobacco Abuse (with respect to "Yes") | 0.94259 | 0.39659 | 2.377 | ***0.0179*** |
| APOE | 0.15661 | 0.35343 | 0.443 | 0.6579 |
| OGG1 | -0.18157 | 0.29956 | -0.606 | 0.5448 |
| Interaction: Cognitive Impairment x Diabetes "Yes" | -0.69414 | 0.79965 | -0.868 | 0.3859 |

| R-squared | 0.1041 | *p*-value | 9.263e-06 |
| --- | --- | --- | --- |
| Adjusted R-squared | 0.07766 | df | 12 and 407 |
| F-statistic | 3.94 | Sample n | 420 |

**Supplementary Table 13.** Cellular 8oxoG “hotspot” variant count multiple linear regression model prediction considering cognitive impairment (NC vs MCI + AD), *OGG1* genotype, and population interaction effect with both sex and years of education within the whole cohort. Italics and bolding indicate a p-value of significance, while italics alone indicate a p-value approaching significance.

| **Variable** | **Coefficient** | **Std. Error** | **t-statistic** | ***p*-value** |
| --- | --- | --- | --- | --- |
| Constant | 2.265021 | 1.24577 | 1.818 | *0.0698* |
| Population (with respect to NHW) | -1.267594 | 0.897224 | -1.413 | 0.1585 |
| Cognitive Impairment | -0.044081 | 0.21078 | -0.209 | 0.8344 |
| Sex (with respect to Male) | -0.212817 | 0.261981 | -0.812 | 0.4171 |
| Age | 0.004656 | 0.014688 | 0.317 | 0.7514 |
| Years of Education | 0.007817 | 0.026768 | 0.292 | 0.7704 |
| BMI | 0.025288 | 0.018028 | 1.403 | 0.1615 |
| Diabetes (with respect to "Yes") | -0.161449 | 0.211734 | -0.763 | 0.4462 |
| Depression (with respect to "Yes") | -0.097161 | 0.260528 | -0.373 | 0.7094 |
| Tobacco Abuse (with respect to "Yes") | 0.205233 | 0.197392 | 1.04 | 0.2991 |
| APOE | 0.085599 | 0.174886 | 0.489 | 0.6248 |
| OGG1 | -0.154501 | 0.148714 | -1.039 | 0.2995 |
| Interaction: NHW x Male "Yes" | 0.417555 | 0.386446 | 1.081 | 0.2806 |
| Interaction: NHW x Years of Education | 0.044535 | 0.059439 | 0.749 | 0.4541 |

| R-squared | 0.02392 | *p*-value | 0.6969 |
| --- | --- | --- | --- |
| Adjusted R-squared | -0.00733 | df | 13 and 406 |
| F-statistic | 0.7655 | Sample n | 420 |

**Supplementary Table 14.** Cellular 8oxoG “hotspot” variant count multiple linear regression model considering cognitive impairment (NC vs. MCI + AD), *OGG1* genotype, and diabetes interaction with cognition. Italics and bolding indicate a p-value of significance, while italics alone indicate a p-value approaching significance.

| **Variable** | **Coefficient** | **Std. Error** | **t-statistic** | ***p*-value** |
| --- | --- | --- | --- | --- |
| Constant | 1.969724 | 1.234291 | 1.596 | 0.111 |
| Population (with respect to NHW) | -0.39896 | 0.239093 | -1.669 | ***0.096*** |
| Cognitive Impairment | 0.094742 | 0.255492 | 0.371 | 0.711 |
| Sex (with respect to Male) | -0.01567 | 0.196599 | -0.08 | 0.937 |
| Age | 0.005782 | 0.014616 | 0.396 | ***6.93e-01*** |
| Years of Education | 0.016958 | 0.02404 | 0.705 | 0.481 |
| BMI | 0.024231 | 0.018065 | 1.341 | 0.181 |
| Diabetes (with respect to "Yes") | 0.046774 | 0.27941 | 0.167 | 0.867 |
| Depression (with respect to "Yes") | -0.05664 | 0.257613 | -0.22 | 0.826 |
| Tobacco Abuse (with respect to "Yes") | 0.181266 | 0.196172 | 0.924 | 0.356 |
| APOE | 0.075946 | 0.174825 | 0.434 | 0.664 |
| OGG1 | -0.15207 | 0.148175 | -1.026 | 0.305 |
| Interaction: Cognitive Impairment x Diabetes "Yes" | -0.43665 | 0.395542 | -1.104 | 0.27 |

| R-squared | 0.02215 | *p*-value | 0.6835 |
| --- | --- | --- | --- |
| Adjusted R-squared | -0.006685 | df | 12 and 407 |
| F-statistic | 0.7681 | Sample n | 420 |

**Supplementary Table 15.** Cellular 8oxoG variant count multiple linear regression prediction model considering cognitive impairment (NC vs. MCI + AD) and OGG1 genotype in the Mexican American population. Italics and bolding indicate a p-value of significance, while italics alone indicate a p-value approaching significance.

| **Variable** | **Coefficient** | **Std. Error** | **t-statistic** | ***p*-value** |
| --- | --- | --- | --- | --- |
| Constant | 5.22225 | 3.65763 | 1.428 | 0.154721 |
| Cognitive Impairment | 1.36712 | 0.64952 | 2.105 | ***0.036397*** |
| Sex (with respect to Male) | -2.28525 | 0.61787 | -3.699 | ***0.000271*** |
| Age | -0.01356 | 0.04394 | -0.308 | 0.757991 |
| Years of Education | 0.15573 | 0.06265 | 2.486 | ***0.013646*** |
| BMI | 0.06814 | 0.05009 | 1.36 | 0.17506 |
| Diabetes (with respect to "Yes") | -0.92301 | 0.61752 | -1.495 | 0.136364 |
| Depression (with respect to "Yes") | -1.4394 | 0.79812 | -1.803 | *0.072626* |
| Tobacco Abuse (with respect to "Yes") | 1.99263 | 0.60204 | 3.31 | ***0.001084*** |
| APOE | 0.21207 | 0.6515 | 0.326 | 0.745087 |
| OGG1 | -0.05146 | 0.42638 | -0.121 | 0.904049 |

| R-squared | 0.1172 | *p*-value | 0.001224 |
| --- | --- | --- | --- |
| Adjusted R-squared | 0.07869 | df | 10 and 229 |
| F-statistic | 3.041 | Sample n | 201 |

**Supplementary Table 16.** Cellular 8oxoG “hotspot” variant count multiple linear regression considering cognitive impairment (NC vs. MCI + AD) and *OGG1* genotype in the Mexican American population. Italics and bolding indicate a p-value of significance, while italics alone indicate a p-value approaching significance.

| **Variable** | **Coefficient** | **Std. Error** | **t-statistic** | ***p*-value** |
| --- | --- | --- | --- | --- |
| Constant | 3.150962 | 1.719516 | 1.832 | *0.0682* |
| Cognitive Impairment | -0.031558 | 0.30535 | -0.103 | 0.9178 |
| Sex (with respect to Male) | -0.340996 | 0.290473 | -1.174 | 0.2416 |
| Age | -0.003081 | 0.020658 | -0.149 | 0.8816 |
| Years of Education | 0.00384 | 0.029454 | 0.13 | 0.8964 |
| BMI | 0.013702 | 0.023548 | 0.582 | 0.5612 |
| Diabetes (with respect to "Yes") | -0.243125 | 0.290305 | -0.837 | 0.4032 |
| Depression (with respect to "Yes") | -0.375051 | 0.37521 | -1 | 0.3186 |
| Tobacco Abuse (with respect to "Yes") | 0.458274 | 0.283027 | 1.619 | 0.1068 |
| APOE | 0.212727 | 0.306281 | 0.695 | 0.488 |
| OGG1 | -0.093201 | 0.200451 | -0.465 | 0.6424 |

| R-squared | 0.02459 | *p*-value | 0.8318 |
| --- | --- | --- | --- |
| Adjusted R-squared | -0.01801 | df | 10 and 229 |
| F-statistic | 0.5773 | Sample n | 201 |

**Supplementary Table 17.** Cellular 8oxoG variant count multiple linear regression predictive model considering cognitive impairment (NC vs. MCI + AD) and *OGG1* genotype in non-Hispanic Whites. Italics and bolding indicate a p-value of significance, while italics alone indicate a p-value approaching significance.

| **Variable** | **Coefficient** | **Std. Error** | **t-statistic** | ***p*-value** |
| --- | --- | --- | --- | --- |
| Constant | 0.513269 | 3.380988 | 0.152 | 0.88 |
| Cognitive Impairment | 0.016123 | 0.495422 | 0.033 | 0.974 |
| Sex (with respect to Male) | 0.604656 | 0.453736 | 1.333 | 0.184 |
| Age | 0.044461 | 0.036718 | 1.211 | 0.228 |
| Years of Education | 0.008396 | 0.083286 | 0.101 | 0.92 |
| BMI | 0.07249 | 0.049341 | 1.469 | 0.144 |
| Diabetes (with respect to "Yes") | -0.175279 | 0.532067 | -0.329 | 0.742 |
| Depression (with respect to "Yes") | -0.02399 | 0.642238 | -0.037 | 0.97 |
| Tobacco Abuse (with respect to "Yes") | -0.14622 | 0.461915 | -0.317 | 0.752 |
| APOE | 0.151415 | 0.362023 | 0.418 | 0.676 |
| OGG1 | -0.29547 | 0.378453 | -0.781 | 0.436 |

| R-squared | 0.03962 | *p*-value | 0.7262 |
| --- | --- | --- | --- |
| Adjusted R-squared | -0.01721 | df | 10 and 169 |
| F-statistic | 0.6971 | Sample n | 180 |

**Supplementary Table 18.** Multiple linear regression predictive model assessing cellular 8oxoG “hotspot” variant count with cognitive impairment (NC vs. MCI + AD) and *OGG1* genotype in non-Hispanic Whites. Italics and bolding indicate a p-value of significance, while italics alone indicate a p-value approaching significance.

| **Variable** | **Coefficient** | **Std. Error** | **t-statistic** | ***p*-value** |
| --- | --- | --- | --- | --- |
| Constant | 0.319717 | 1.9925 | 0.16 | 0.873 |
| Cognitive Impairment | 0.080826 | 0.291964 | 0.277 | 0.782 |
| Sex (with respect to Male) | 0.192608 | 0.267398 | 0.72 | 0.472 |
| Age | 0.008598 | 0.021639 | 0.397 | 0.692 |
| Years of Education | 0.05812 | 0.049082 | 1.184 | 0.238 |
| BMI | 0.042181 | 0.029078 | 1.451 | 0.149 |
| Diabetes (with respect to "Yes") | -0.093654 | 0.31356 | -0.299 | 0.766 |
| Depression (with respect to "Yes") | 0.318328 | 0.378487 | 0.841 | 0.402 |
| Tobacco Abuse (with respect to "Yes") | -0.121255 | 0.272218 | -0.445 | 0.657 |
| APOE | -0.103619 | 0.213349 | -0.486 | 0.628 |
| OGG1 | -0.293728 | 0.223032 | -1.317 | 0.19 |

| R-squared | 0.04369 | *p*-value | 0.6556 |
| --- | --- | --- | --- |
| Adjusted R-squared | -0.0129 | df | 10 and 169 |
| F-statistic | 0.772 | Sample n | 180 |

**Assessment of ccf-mtDNA 8oxoG Variant Count in MA and NHW TARCC Participants**

**Supplementary Table 19.** *APOE* genotype directly sequenced vs. imputed in plasma of TARCC participants by population.

| **Non-Hispanic Whites** | **N = 65** |
| --- | --- |
| *APOE* Genotyped | (89%) |
| *APOE* Imputed | (0%) |
| *APOE* Missing | (11%) |
| **Mexican Americans** | **N = 57** |
| *APOE* Genotyped | (98%) |
| *APOE* Imputed | (2%) |
| *APOE* Missing | (0%) |

**Supplementary Table 20.** *APOE* and *OGG1* genotype frequencies in each population based on cognitive phenotype in TARCC participants assessed via plasma.

| **Non-Hispanic Whites** | | **NC** | **AD** |
| --- | --- | --- | --- |
| *APOE* Genotype  (n = 58) | ε2/ε2 | 0 (0%) | 0 (0%) |
|  | ε2/ε3 | 2 (6.1%) | 0 (0%) |
|  | ε2/ε4 | 1 (3%) | 0 (0%) |
|  | ε3/ε3 | 19 (57.6%) | 8 (25%) |
|  | ε3/ε4 | 5 (15.2%) | 16 (50%) |
|  | ε4/ε4 | 0 (0%) | 7 (21.9%) |
|  |  |  |  |
| *OGG1* Genotype  (n = 43) | Ser326 | 10 (30.3%) | 15 (46.9%) |
|  | Ser/Cys326 | 8 (24.2%) | 7 (21.9%) |
|  | Cys326 | 2 (6.1%) | 1 (3.1%) |
|  |  |  |  |
| **Mexican Americans** | | **NC** | **AD** |
| *APOE* Genotype  (n = 57) | ε2/ε2 | 0 (0%) | 0 (0%) |
|  | ε2/ε3 | 3 (10%) | 0 (0%) |
|  | ε2/ε4 | 0 (0%) | 0 (0%) |
|  | ε3/ε3 | 22 (73.3%) | 15 (26.3%) |
|  | ε3/ε4 | 5 (16.7%) | 12 (44.4%) |
|  | ε4/ε4 | 0 (0%) | 0 (0%) |
|  |  |  |  |
| *OGG1* Genotype  (n = 50) | Ser326 | 5 (16.7%) | 9 (33.3%) |
|  | Ser/Cys326 | 20 (66.7%) | 9 (33.3%) |
|  | Cys326 | 3 (10%) | 4 (14.8%) |

**Supplementary Table 21.** Hardy-Weinberg check for *APOE* in plasma subset of TARCC participants.

|  | **Total Cohort (N = 115)** | **Non-Hispanic Whites (N = 58)** | **Mexican Americans (N = 57)** |
| --- | --- | --- | --- |
| ε2 | 0.02609 | 0.02586 | 0.02632 |
| ε3 | 0.7435 | 0.6638 | 0.8246 |
| ε4 | 0.2304 | 0.3103 | 0.1491 |
| Observed ε2/ε2 | 0.00 | 0.00 | 0.00 |
| Observed ε2/ε3 | 0.04348 | 0.03448 | 0.05263 |
| Observed ε2/ε4 | 0.008696 | 0.01724 | 0.00 |
| Observed ε3/ε3 | 0.5565 | 0.4655 | 0.6491 |
| Observed ε3/ε4 | 0.3304 | 0.3621 | 0.2982 |
| Observed ε4/ε4 | 0.06087 | 0.1207 | 0.00 |
| Expected ε2/ε2 | 0.0006805 | 0.0006689 | 0.0006925 |
| Expected ε2/ε3 | 0.03879 | 0.03433 | 0.04340 |
| Expected ε2/ε4 | 0.01202 | 0.01605 | 0.007849 |
| Expected ε3/ε3 | 0.5528 | 0.4406 | 0.6799 |
| Expected ε3/ε4 | 0.3426 | 0.4120 | 0.2459 |
| Expected ε4/ε4 | 0.05310 | 0.09631 | 0.02224 |
| ***p*-value** | ***0.9981*** | ***0.9928*** | ***0.9776*** |

**Supplementary Table 22.** Hardy-Weinberg check for *OGG1* in plasma subset of TARCC participants.

|  | **Total Cohort (N = 93)** | **Non-Hispanic Whites (N = 43)** | **Mexican Americans (N = 50)** |
| --- | --- | --- | --- |
| Ser326 | 0.6559 | 0.7558 | 0.5700 |
| Cys326 | 0.3441 | 0.2442 | 0.4300 |
| Observed Ser326/Ser326 | 0.4194 | 0.5814 | 0.2800 |
| Observed Ser326/Cys326 | 0.4731 | 0.3488 | 0.5800 |
| Observed Cys326/Cys326 | 0.1075 | 0.06978 | 0.1400 |
| Expected Ser326/Ser326 | 0.4302 | 0.5713 | 0.3249 |
| Expected Ser326/Cys326 | 0.4514 | 0.3691 | 0.4902 |
| Expected Cys326/Cys326 | 0.1184 | 0.05963 | 0.1849 |
| ***p*-value** | ***0.9616*** | ***0.9562*** | ***0.8546*** |


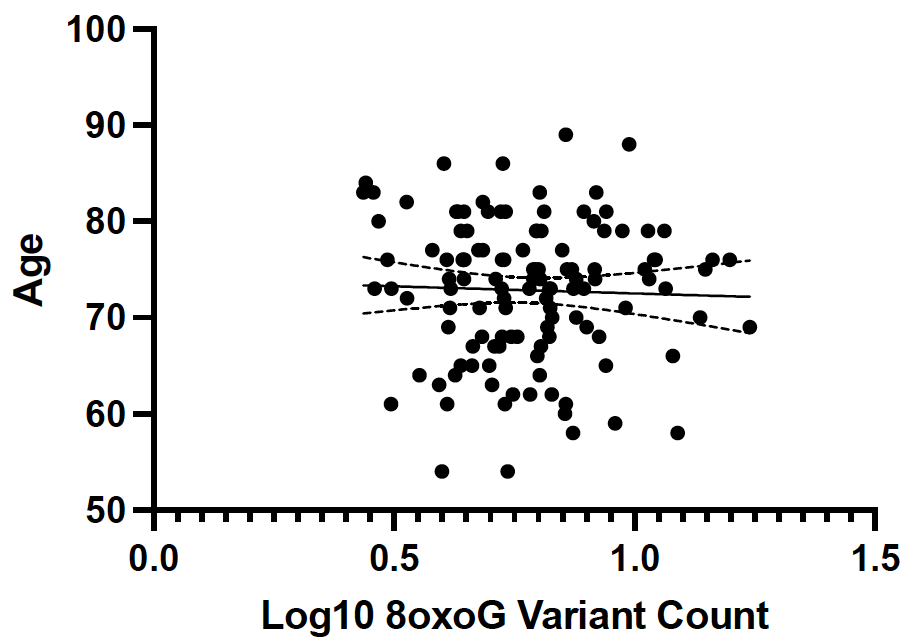


**Supplementary Figure 3.**  **Scatter plot of log 8oxoG variant count by age.** Sample means of total log transformed plasma 8oxoG variant count was assessed by age using a two-tailed Pearson correlation (n = 122). Dotted lines represent 95% confidence interval (-0.2113 to 0.1438), and the solid line indicates the best-fit line. Correlation statistics: r = -0.03489, R squared = 0.001217, p-value = 0.7028.

**Supplementary Figure 4.** **ccf-mtDNA 8oxoG variant count is not significantly associated with cognitive status in the whole cohort.** Log transformed ccf-mtDNA 8oxoG variant count by cognitive phenotype was analyzed via an unpaired two-tailed t-test (*n* = 122, *t*-statistic = 0.6647, df = 120, *p* = 0.5075). Black filled circles represent individual data points and error bars represent standard error of the mean. Effect size and confident interval plotted on right y-axis. The triangle represents the difference of the means.

**Supplementary Figure 5. ccf-mtDNA 8oxoG variant count does not significantly differ by sex in the whole cohort.** Log transformed ccf-mtDNA 8oxoG variant count grouped by sex via unpaired, two-tailed t-test (*n* = 122, *t*-statistic = 0.6705, df = 120, *p* = 0.5039). Circles indicate individual points and error bars are representative of the standard error of the mean. Effect size and confident interval plotted on right y-axis. The triangle represents the difference of the means.

**Supplementary Table 23.** Multiple linear regression predictive model for ccf-mtDNA 8oxoG variant count by cognitive status considering a population interaction with sex and education in the whole cohort. Italics and bolding indicate a p-value of significance, while italics alone indicate a p-value approaching significance.

| **Variable** | **Coefficient** | **Std. Error** | **t-statistic** | **p-value** |
| --- | --- | --- | --- | --- |
| Constant | 1.1077504 | 0.2272524 | 4.875 | ***5.53E-06*** |
| Population with respect to NHW | -0.1377608 | 0.137145 | -1.004 | 0.3182 |
| Cognitive Status with respect to AD | -0.060558 | 0.0375285 | -1.614 | 0.1106 |
| Sex with respect to Male | -0.055037 | 0.0454276 | -1.212 | 0.2293 |
| Age | -0.0034511 | 0.002417 | -1.428 | 0.1573 |
| Years of Education | -0.0006932 | 0.0045778 | -0.151 | 0.88 |
| BMI | 0.0021872 | 0.003657 | 0.598 | 0.5515 |
| Diabetes with respect to "Yes" | -0.0564732 | 0.03651 | -1.547 | 0.1259 |
| Depression (with respect to "Yes") | 0.0082359 | 0.0392742 | 0.21 | 0.8344 |
| Tobacco Abuse (with respect to "Yes") | -0.0398914 | 0.0338296 | -1.179 | 0.2419 |
| APOE | 0.0792337 | 0.0317264 | 2.497 | ***0.0146*** |
| OGG1 | 0.0078175 | 0.0258873 | 0.302 | 0.7635 |
| Interaction: NHW x Male "Yes" | 0.1613152 | 0.0651314 | 2.477 | ***0.0154*** |
| Interaction: NHW x Years of Education | -0.0069258 | 0.0098316 | -0.704 | 0.4832 |

| R-squared | 0.3614 | *p*-value | 0.0003131 |
| --- | --- | --- | --- |
| Adjusted R-squared | 0.2564 | df | 13 and 79 |
| F-statistic | 3.44 | Sample n | 93 |

**Supplementary Table 24.** ccf-8oxoG variant count and cognitive status (NC vs AD) multiple linear regression model prediction considering diabetes interaction effect with AD. Italics and bolding indicate a p-value of significance, while italics alone indicate a p-value approaching significance.

| **Variable** | **Coefficient** | **Std. Error** | **t-statistic** | **p-value** |
| --- | --- | --- | --- | --- |
| Constant | 1.04047 | 0.234997 | 4.428 | ***2.99E-05*** |
| Population with respect to NHW | -0.155877 | 0.041492 | -3.757 | ***0.000325*** |
| Cognitive Status with respect to AD | -0.041655 | 0.047264 | -0.881 | 0.380786 |
| Sex with respect to Male | 0.021156 | 0.034584 | 0.612 | 0.542458 |
| Age | -0.002633 | 0.002471 | -1.066 | 0.28968 |
| Years of Education | -0.002855 | 0.004297 | -0.664 | 0.508323 |
| BMI | 0.002166 | 0.003781 | 0.573 | 0.568403 |
| Diabetes with respect to "Yes" | -0.04054 | 0.053793 | -0.754 | 0.453285 |
| Depression (with respect to "Yes") | 0.010261 | 0.040097 | 0.256 | 0.798686 |
| Tobacco Abuse (with respect to "Yes") | -0.047004 | 0.034629 | -1.357 | 0.178483 |
| APOE | 0.0698 | 0.032464 | 2.15 | ***0.034571*** |
| OGG1 | -0.001137 | 0.026537 | -0.043 | 0.965928 |
| Interaction: AD x Diabetes "Yes" | -0.040233 | 0.070816 | -0.568 | 0.571531 |

| R-squared | 0.3121 | *p*-value | 0.001531 |
| --- | --- | --- | --- |
| Adjusted R-squared | 0.2089 | df | 12 and 80 |
| F-statistic | 3.025 | Sample n | 93 |

**
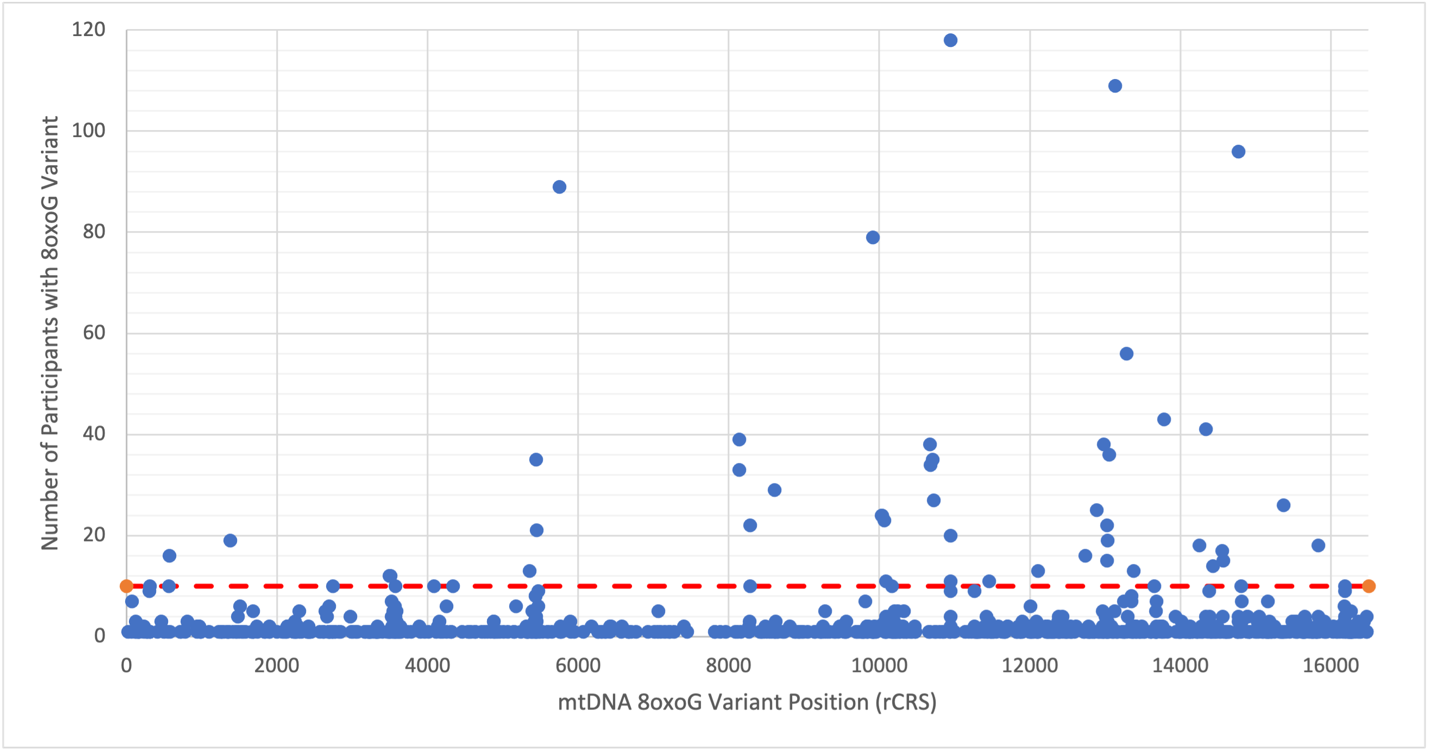
**

**Supplementary Figure 6.** Distribution of ccf-8oxoG variants within the mitochondrial genome by number of individuals with 8oxoG variant for 8oxoG “hotspot” variant selection. The red dashed line intercepts at 25 subjects with the 8oxoG variant at the specified position. 8oxoG variants above the intercept were conveniently selected as “hotspots”.

**Supplementary Table 25.** ccf-8oxoG “hotspot” variant count multiple linear regression model prediction considering cognitive status (NC vs AD) and population interaction effect with both sex and education. Italics and bolding indicate a p-value of significance, while italics alone indicate a p-value approaching significance.

| **Variable** | **Coefficient** | **Std. Error** | **t-statistic** | ***p*-value** |
| --- | --- | --- | --- | --- |
| Constant | 0.5294717 | 0.2118443 | 2.499 | ***0.0145*** |
| Population (with respect to NHW) | -0.0082257 | 0.1278463 | -0.064 | 0.9489 |
| Cognitive Status (with respect to AD) | -0.0465321 | 0.034984 | -1.33 | 0.1873 |
| Sex (with respect to Male) | -0.0150998 | 0.0423475 | -0.357 | 0.7224 |
| Age | -0.0036469 | 0.0022531 | -1.619 | 0.1095 |
| Years of Education | 0.0025028 | 0.0042674 | 0.586 | 0.5592 |
| BMI | 0.0028833 | 0.0034091 | 0.846 | 0.4002 |
| Diabetes (with respect to "Yes") | 0.0169052 | 0.0340345 | 0.497 | 0.6208 |
| Depression (with respect to "Yes") | -0.0006082 | 0.0366113 | -0.017 | 0.9868 |
| Tobacco Abuse (with respect to "Yes") | 0.0248699 | 0.0315359 | 0.789 | 0.4327 |
| APOE | 0.0118648 | 0.0295753 | 0.401 | 0.6894 |
| OGG1 | -0.0067661 | 0.0241321 | -0.28 | 0.7799 |
| Interaction: NHW x Male "Yes" | 0.0292928 | 0.0607154 | 0.482 | 0.6308 |
| Interaction: NHW x Years of Education | -0.0027096 | 0.009165 | -0.296 | 7.68e-01 |

| R-squared | 0.09905 | *p*-value | 0.7879 |
| --- | --- | --- | --- |
| Adjusted R-squared | -0.04921 | df | 13 and 79 |
| F-statistic | 0.6681 | Sample n | 93 |

**Supplementary Table 26.** ccf-8oxoG “hotspot” variant count multiple linear regression model prediction considering cognitive status (NC vs AD) and diabetes interaction effect with cognition. Italics and bolding indicate a p-value of significance, while italics alone indicate a p-value approaching significance.

| **Variable** | **Coefficient** | **Std. Error** | **t-statistic** | ***p*-value** |
| --- | --- | --- | --- | --- |
| Constant | 0.487273 | 0.210374 | 2.316 | ***0.0231*** |
| Population (with respect to NHW) | -0.032477 | 0.037144 | -0.874 | 0.3845 |
| Cognitive Status (with respect to AD) | -0.023605 | 0.042312 | -0.558 | 0.5785 |
| Sex (with respect to Male) | -0.002295 | 0.03096 | -0.074 | 0.9411 |
| Age | -0.003352 | 0.002212 | -1.516 | 0.1335 |
| Years of Education | 0.002646 | 0.003847 | 0.688 | 0.4936 |
| BMI | 0.003128 | 0.003385 | 0.924 | 0.3582 |
| Diabetes (with respect to "Yes") | 0.046835 | 0.048157 | 0.973 | 0.3337 |
| Depression (with respect to "Yes") | -0.002148 | 0.035895 | -0.06 | 0.9524 |
| Tobacco Abuse (with respect to "Yes") | 0.022069 | 0.031001 | 0.712 | 0.4786 |
| APOE | 0.009159 | 0.029063 | 0.315 | 0.7535 |
| OGG1 | -0.00954 | 0.023756 | -0.402 | 0.6891 |
| Interaction: Cognitive Impairment x Diabetes "Yes" | -0.05787 | 0.063396 | -0.913 | 3.64e-01 |

| R-squared | 0.1049 | *p*-value | 0.6679 |
| --- | --- | --- | --- |
| Adjusted R-squared | -0.02937 | df | 12 and 80 |
| F-statistic | 0.7813 | Sample n | 93 |

**Supplementary Table 27.** ccf-8oxoG “hotspot” variant count multiple linear regression considering cognitive status (NC vs. AD) and *OGG1* genotype in the Mexican American population. Italics and bolding indicate a p-value of significance, while italics alone indicate a p-value approaching significance.

| **Variable** | **Coefficient** | **Std. Error** | **t-statistic** | ***p*-value** |
| --- | --- | --- | --- | --- |
| Constant | 0.1634 | 0.2478 | 0.659 | 0.514 |
| Cognitive Impairment | -0.02514 | 0.0422 | -0.596 | 0.555 |
| Sex (with respect to Male) | -0.01103 | 0.03924 | -0.281 | 0.78 |
| Age | 2.358e-05 | 0.002607 | 0.009 | 0.993 |
| Years of Education | 0.002524 | 0.004078 | 0.619 | 0.54 |
| BMI | 0.005411 | 0.004145 | 1.306 | 0.199 |
| Diabetes (with respect to "Yes") | 0.06851 | 0.04369 | 1.568 | 0.125 |
| Depression (with respect to "Yes") | -0.03252 | 0.05121 | -0.635 | 0.529 |
| Tobacco Abuse (with respect to "Yes") | -0.003593 | 0.04178 | -0.086 | 0.932 |
| APOE | -0.02626 | 0.04836 | -0.543 | 0.59 |
| OGG1 | 0.01217 | 0.03022 | 0.403 | 0.689 |

| R-squared | 0.1937 | *p*-value | 0.5113 |
| --- | --- | --- | --- |
| Adjusted R-squared | -0.01309 | df | 10 and 39 |
| F-statistic | 0.9367 | Sample n | 50 |

**Supplementary Table 28.** Multiple linear regression predictive model for ccf-mtDNA 8oxoG variant count by cognitive status (NC vs. AD) and *OGG1* genotype in non-Hispanic Whites. Italics and bolding indicate a p-value of significance, while italics alone indicate a p-value approaching significance.

| **Variable** | **Coefficient** | **Std. Error** | **t-statistic** | ***p*-value** |
| --- | --- | --- | --- | --- |
| Constant | 1.073163 | 0.370779 | 2.894 | ***0.00679*** |
| Cognitive Impairment | -0.054296 | 0.060108 | -0.903 | 0.37311 |
| Sex (with respect to Male) | 0.036008 | 0.053041 | 0.679 | 0.5021 |
| Age | -0.009379 | 0.004213 | -2.226 | ***0.03316*** |
| Years of Education | 0.001405 | 0.009377 | 0.15 | 0.88185 |
| BMI | -0.003678 | 0.006274 | -0.586 | 0.56186 |
| Diabetes (with respect to "Yes") | -0.017384 | 0.058084 | -0.299 | 0.76665 |
| Depression (with respect to "Yes") | 0.038942 | 0.055116 | 0.707 | 0.48496 |
| Tobacco Abuse (with respect to "Yes") | 0.016803 | 0.050551 | 0.332 | 0.74175 |
| APOE | 0.028238 | 0.045145 | 0.625 | 0.53608 |
| OGG1 | 0.012642 | 0.04151 | 0.305 | 0.76267 |

| R-squared | 0.1828 | *p*-value | 0.7035 |
| --- | --- | --- | --- |
| Adjusted R-squared | -0.07258 | df | 10 and 32 |
| F-statistic | 0.7158 | Sample n | 43 |
